# FOXA3 regulates cholesterol metabolism to compensate for low uptake during the progression of lung adenocarcinoma

**DOI:** 10.1371/journal.pbio.3002621

**Published:** 2024-05-28

**Authors:** Dongmei Wang, Yuxiang Cao, Meiyao Meng, Jin Qiu, Chao Ni, Xiaozhen Guo, Yu Li, Shuang Liu, Jian Yu, Mingwei Guo, Jiawen Wang, Bing Du, Wenwei Qiu, Cen Xie, Bing Zhao, Xinran Ma, Xinghua Cheng, Lingyan Xu

**Affiliations:** 1 Shanghai Key Laboratory of Regulatory Biology, Institute of Biomedical Sciences and School of Life Sciences, East China Normal University, Shanghai, China; 2 Department of Gastrointestinal Surgery, the Affiliated Changzhou, No. 2 People’s Hospital of Nanjing Medical University, Changzhou Medical Center, Nanjing Medical University, Changzhou, Jiangsu, China; 3 Institute of Organoid Technology, BioGenous Biotechnology, Inc., Suzhou, China; 4 State Key Laboratory of Drug Research, Shanghai Institute of Materia Medica, Chinese Academy of Sciences, Shanghai, China; 5 Joint Center for Translational Medicine, Fengxian District Central Hospital, Fengxian District, Shanghai, China; 6 Shanghai Engineering Research Center of Molecular Therapeutics and New Drug Development, School of Chemistry and Molecular Engineering, East China Normal University, Shanghai, China; 7 School of Basic Medical Sciences, Jiangxi Medical College, Nanchang University, Nanchang, Jiangxi, China; 8 Chongqing Key Laboratory of Precision Optics, Chongqing Institute of East China Normal University, Chongqing, China; 9 Department of Oncology, Shanghai Lung Cancer Center, Shanghai Chest Hospital, Shanghai Jiaotong University, Shanghai, China; University of California Los Angeles, UNITED STATES

## Abstract

Cholesterol metabolism is vital for multiple cancer progression, while how cholesterol affects lung, a low-cholesterol tissue, for cancer metastasis and the underlying mechanism remain unclear. In this study, we found that metastatic lung adenocarcinoma cells acquire cellular dehydrocholesterol and cholesterol by endogenous cholesterol biosynthesis, instead of uptake upon cholesterol treatment. Besides, we demonstrated that exogenous cholesterol functions as signaling molecule to induce FOXA3, a key transcription factor for lipid metabolism via GLI2. Subsequently, ChIP-seq analysis and molecular studies revealed that FOXA3 transcriptionally activated Hmgcs1, an essential enzyme of cholesterol biosynthesis, to induce endogenous dehydrocholesterol and cholesterol level for membrane composition change and cell migration. Conversely, FOXA3 knockdown or knockout blocked cholesterol biosynthesis and lung adenocarcinoma metastasis in mice. In addition, the potent FOXA3 inhibitor magnolol suppressed metastatic gene programs in lung adenocarcinoma patient-derived organoids (PDOs). Altogether, our findings shed light onto unique cholesterol metabolism and FOXA3 contribution to lung adenocarcinoma metastasis.

## Abstract 2

Cholesterol is important for cancer progression, yet its levels are low in the lung. How is this overcome during lung cancer? This study shows that exogenous cholesterol can be sensed by lung adenocarcinoma cells, leading to activation of de novo cholesterol biosynthesis through a GLI2-FOXA3-HMGCS1 transcriptional axis, thereby promoting cancer progression.

## Introduction

Lung adenocarcinoma is characterized of strong metastatic capacity, thus is associated with poor prognosis and becomes one of the leading causes of mortality in cancer patients [1]. Cancer cells undergo extensive metabolic reprogramming to fulfill the energy requirements and cope with the foreign hostile environments of the metastatic process. For example, the proper rewiring of lipid metabolism in metastatic cells is critical in providing various lipids as fuel or as signal molecules to fulfill their heightened needs of growth and signal transduction [2].

Cholesterol is a major lipid species. Cholesterol is fundamental components of cellular and organelle membranes and synthetic sources of hormones and bile acid [3]. Intracellular cholesterol is either de novo biosynthesized from Acetyl-CoA via key enzymes such as 3-Hydroxy-3-Methylglutaryl-CoA Synthase 1 (HMGCS1) and 3-Hydroxy-3-Methylglutaryl-CoA Reductase (HMGCR) under the regulation of SREBP2, or taken up from the bloodstream in the form of low-density lipoprotein (LDL) via LDL receptor [4]. Cholesterol plays important roles during cancer progression. For example, cholesterol directly binds to and covalently modifies Hedgehog (Hh) or SMO proteins, thus serves as a signaling molecular to modulate hedgehog signaling pathway in tissue development and tumorigenesis [5,6]. Besides, cholesterol is enriched in patches of cell membrane enriched with membrane proteins, which are essential to mediate the active membrane trafficking and signal transduction during cancer development [7–9]. Intriguingly, cholesterol’s role in cancer development seems to be complexed, as it has been reported that cholesterol and its metabolites including bile acids and oxysterols directly support colon and prostate cancers progression [10], while on the other hand, cholesterol pathway inhibition promotes metastasis and is associated with poor prognosis in pancreatic cancer [11]. Furthermore, cholesterol sometimes plays opposite roles in cancer development in both hepatocarcinoma and breast cancers [12–15]. All these evidences suggest the complexity of cholesterol metabolism during cancer progression, which may differ under different cell types including immune cells and cancer subtype contexts, or by engaging different cholesterol downstream signaling pathways [16].

Importantly, compared to the lipid-rich, cholesterol-rich organs like mammary gland or liver, the cholesterol contents and its regulation in lung are not well understood. The cholesterol content in lung epithelial cells is strictly regulated since excessive cholesterol may cause pathological changes in lung, i.e., cholesterol pneumonia [17]. Dietary cholesterol has been implicated to promote lung cancer [18], yet the roles and mechanisms of cholesterol in lung cancers remain largely unknown. Previous lipidomic analysis revealed that there is significant accumulation of free cholesterol and cholesteryl esters within lung adenocarcinoma [19]. However, it is not clear how cholesterol accumulates in lung adenocarcinoma in face of the tightly regulated cholesterol levels in lung, how cholesterol affects lung adenocarcinoma development and its intrinsic mechanism.

Forkhead box A3 (FOXA3) acts as a transcription factor for direct transcriptional regulation or as a pioneer factor to open the compacted chromatin for transcription activation [20]. We and others have shown that FOXA3 is a key regulator of lipid and glucose metabolism in adipose tissues and in liver under physiological and pathological conditions such as high-fat diet, fasting, glucocorticoid signaling, aging, and hepatic ER stress [21–25]. Meanwhile, it has been reported that FOXA3 is highly expressed in lung adenocarcinoma [26] and Foxa3 overexpression in airway epithelial cells may increase susceptibility to viral infections-associated chronic lung disorders such as chronic goblet cell metaplasia [27], suggesting the involvement of FOXA3 in lung physiology. However, whether FOXA3 is involved in lipid metabolic reprogramming in lung adenocarcinoma metastasis and the underlying mechanism remain to be determined.

In the present study, we demonstrated that exogenous cholesterol functions as a signal molecule in metastatic lung adenocarcinoma and activates de novo cholesterol biosynthesis through the GLI2-FOXA3-HMGCS1 transcriptional axis, which results in increased levels of dehydrocholesterol and cholesterol, promoted membrane composition change, and increased metastasis in lung adenocarcinoma. In addition, reduction of FOXA3 or HMGCS1 suppressed cholesterol and dehydrocholesterol levels and decreased metastatic gene programs in lung adenocarcinoma patient-derived organoids (PDOs). Overall, our results may provide novel therapeutic guidelines for cancer intervention in clinic, which highlighted the importance of blocking de novo cholesterol biosynthesis instead of limiting dietary cholesterol in cancers feature low-cholesterol uptake, i.e., lung adenocarcinoma.

## Results

### Exogenous cholesterol promoted de novo biosynthesis and cancer cell migration in lung adenocarcinoma cells

**Fig 1:**
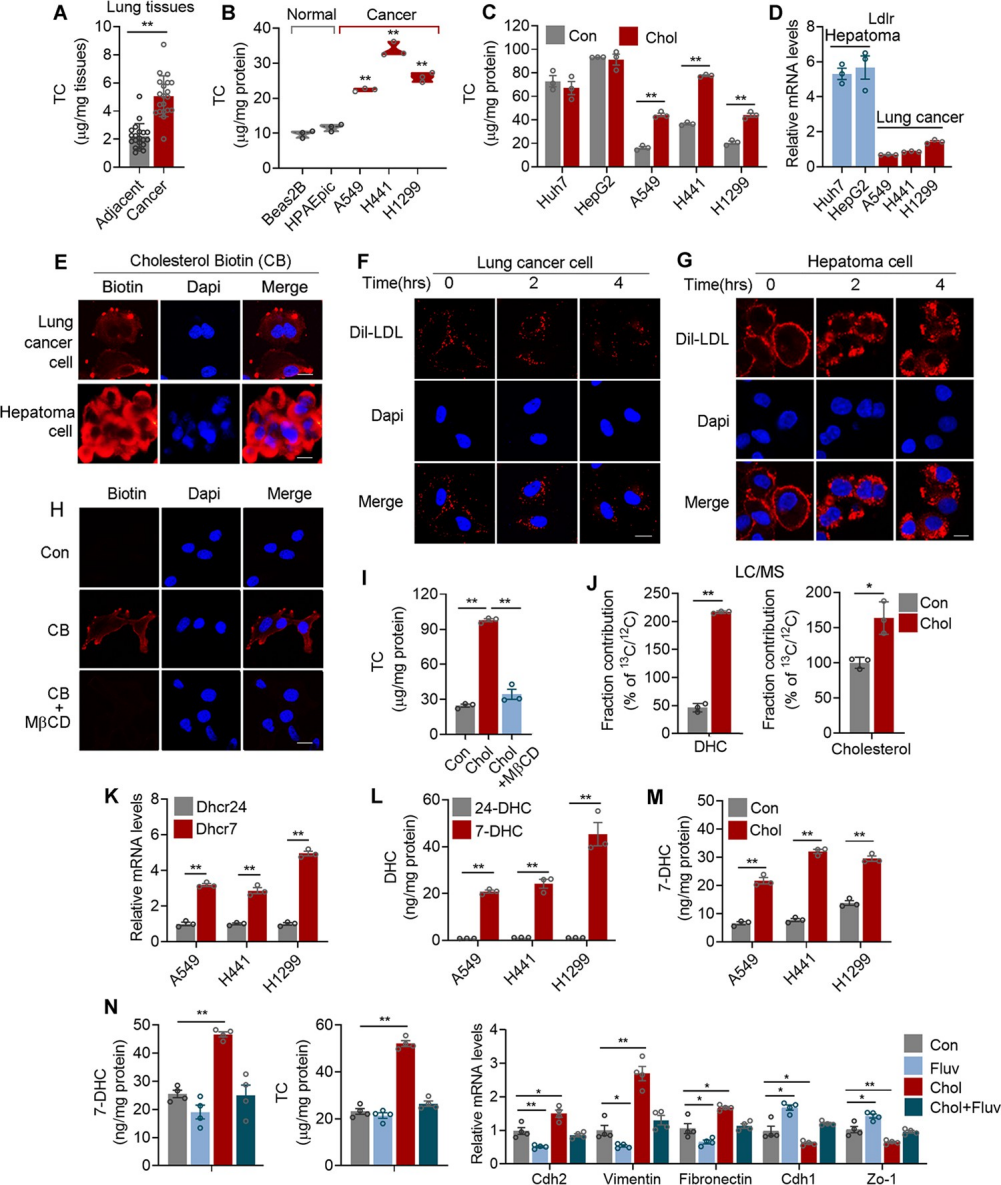
Lung adenocarcinoma cells sense exogenous cholesterol as signal and acquire cellular cholesterol via de novo biosynthesis, instead of uptake, for cell migration. **(A)** The cholesterol levels of human lung adenocarcinoma and adjacent lung tissues (*n* = 20). **(B)** The cholesterol levels of lung cancer and normal epithelial cells (*n* = 3). **(C, D)** The cholesterol **(C)** and qPCR of Ldlr mRNA **(D)** levels of hepatoma and lung cancer cells treated with 5 μg/ml cholesterol for 24 h in 5% LPDS medium (RMPI1640 containing 5% LPDS, *n* = 3). **(E)** Cholesterol biotin (CB) uptake in A549 or HepG2 cells examined by immunofluorescent staining after cells were treated with 5 μg/ml CB cultured in 5% LPDS medium for 24 h. **(F, G)** Dil-LDL uptake of A549 **(F)** or HepG2 **(G)** cells treated with 10 μg/ml Dil-LDL cultured in 5% LPDS medium for 24 h examined by immunofluorescent staining. **(H)** CB uptake in A549 or HepG2 cells with or without MβCD pretreatment compared to untreated cells examined by immunofluorescent staining. **(I)** The cholesterol levels of A549 cells treated with 5 μg/ml cholesterol with or without MβCD pretreatment cultured in LPDS medium for 24 h (*n* = 3). **(J)** LC-MS analysis of ^13^C-acetate-derived ^13^C fractional contribution in cholesterol and dehydrocholesterol extracted from A549 cells treated with 5 μg/ml cholesterol cultured in LPDS medium (*n* = 3). **(K)** qRT-PCR analyses of Dhcr7 and Dhcr24 of lung cancer cells (*n* = 3). **(L)** The 7-dehydrocholesterol (7-DHC) and 24-dehydrocholesterol (24-DHC) levels of lung cancer cells were analyzed by ELISA kits (*n* = 3). **(M)** The 7-DHC levels of lung cancers cultured in LPDS medium treated with or without 5 μg/ml Cholesterol for 24 h detected by ELISA kit (*n* = 3). **(N**) The 7-DHC, cholesterol levels, and qRT-PCR analyses of metastatic-related genes of A549 cells cultured in LPDS medium treated with 5 μg/ml cholesterol with or without 10 μm fluvastatin for 24 h (*n* = 4). Scale bars, 20 μm (E–H). Data were presented as mean ± SEM. *, *P* < 0.05; **, *P* < 0.01. LPDS, lipoprotein deficient serum; CB, Cholesterol biotin; DiI-LDL, Human DiI-Low Density Lipoprotein; TC, total cholesterol; 7-DHC, 7-dehydrocholesterol; 24-DHC, 24-dehydrocholesterol; MβCD, methyl-β-cyclodextrin. The data underlying this figure can be found in the Supporting information file S1 Data.

Lung tissue is a cholesterol-low tissue compared to breast, brain, and liver tissues (S1A Fig). However, cholesterol levels of lung adenocarcinoma were significantly higher than that of adjacent lung tissues (Fig 1A). We observed similar features in in vitro lung adenocarcinoma cellular models, which showed significantly higher cholesterol levels compared to normal lung epithelial cells (Fig 1B), suggesting the involvement of cholesterol metabolism in lung cancer.

To understand the role of cholesterol in lung cancer development, we treated lung cancer cell lines with cholesterol and compared their responses to the typical high cholesterol tumor hepatoma cells. We found that cholesterol addition did not increase, even slightly suppressed the intracellular cholesterol in hepatoma cells (Fig 1C), which is in consistent with previous reports of an inhibitory role of extracellular cholesterol uptake on cholesterol synthesis in liver cancers [28]. In contrast, cholesterol treatment significantly enhanced intracellular cholesterol levels in lung cancer cells (Fig 1C), accompanied with elevated metastatic gene programs at both mRNA and protein levels, as well as higher migration capacities (S1B–S1D Fig), without obvious influence on cell proliferation (S1E Fig).

To understand why exogenous cholesterol treatment increased intracellular cholesterol synthesis in lung adenocarcinoma cells but not in hepatoma cells, we further treated lung adenocarcinoma cells with biotin-labeled cholesterol (CB) to trace the fate of exogenous cholesterol and comparing it with hepatoma cells [6]. We observed that CB were predominantly retained on cell membranes of lung cancer cells, in sharp contrast to hepatoma cells wherein CB were actively internalized into cell plasma (Figs 1E and S1F). Consistent with this, we found significantly lower expression of cholesterol receptor LDL receptor (LDLR) in lung adenocarcinoma cells compared to hepatoma cells (Fig 1D). Cholesterol is mainly transported and taken up by cells in LDL particles via LDLR. Similarly, we found that florescent LDL (Dil-LDL) were still retained on membranes in lung cancer cells 4 h after treatment (Figs 1F and S1G), while Dil-LDL were notably taken up into cytoplasm in hepatoma cells in a time-dependent manner (Figs 1G and S1G).

Cholesterol has been shown to function as a signal molecule by binding to and modifying factors in Hedgehog signaling pathway enriched on cell membrane [29]. Interfering membrane signal transduction in lung cancer cells with Methyl-β-Cyclodextrin (MβCD), a substance depleting membrane cholesterol [30] (S1H Fig), we found that the subsequent administration of biotin-labeled cholesterol no longer bound to membrane and failed to increase cellular cholesterol levels in lung adenocarcinoma cells (Fig 1H and 1I). These results suggested that exogenous cholesterol may induce different signals in lung adenocarcinoma cells or in hepatoma cells, one involving membrane signaling while one engaging cytosolic cascade. Critically, we treated lung adenocarcinoma cells with ^13^C-acetate tracer and performed metabolic flux analysis. Subsequent ^13^C tracing indicated that exogenous cholesterol potently activated de novo cholesterol synthesis in lung cancer, as evident by the significant increases in ^13^C-dehydrocholesterol (DHC) and ^13^C-cholesterol in treated group compared to control (Fig 1J).

The last step of de novo cholesterol synthesis is the conversion of 24-dehydrocholesterol (24-DHC) or 7-dehydrocholesterol (7-DHC) into cholesterol, catalyzed by 24-Dehydrocholesterol Reductase (DHCR24) or 7-Dehydrocholesterol Reductase (DHCR7), respectively [31]. Interestingly, we found significantly higher mRNA level of DHCR7 compared to DHCR24, while consistently, 7-DHC is the dominant form of cholesterol precursor in lung adenocarcinoma cells (Fig 1K and 1L). Cholesterol treatment significantly increased 7-DHC levels in lung cancer cells (Fig 1M). Fluvastatin inhibits cholesterol biosynthesis [32]. We found that exogenous cholesterol-induced increase in intracellular 7-DHC and cholesterol levels, as well as metastatic gene programs expression (Fig 1N) in lung cancer cells were blunted by Fluvastatin treatment.

Overall, these data indicated that exogenous cholesterol promoted cell migration by potently activating de novo cholesterol synthesis and inducing 7-DHC and cholesterol levels in lung adenocarcinoma, possibly as a way to circumvent the typically low-cholesterol levels in lung tissues.

### FOXA3 is a key transcriptional factor to mediate extracellular cholesterol-induced de novo cholesterol biosynthesis in lung adenocarcinoma cells

**Fig 2:**
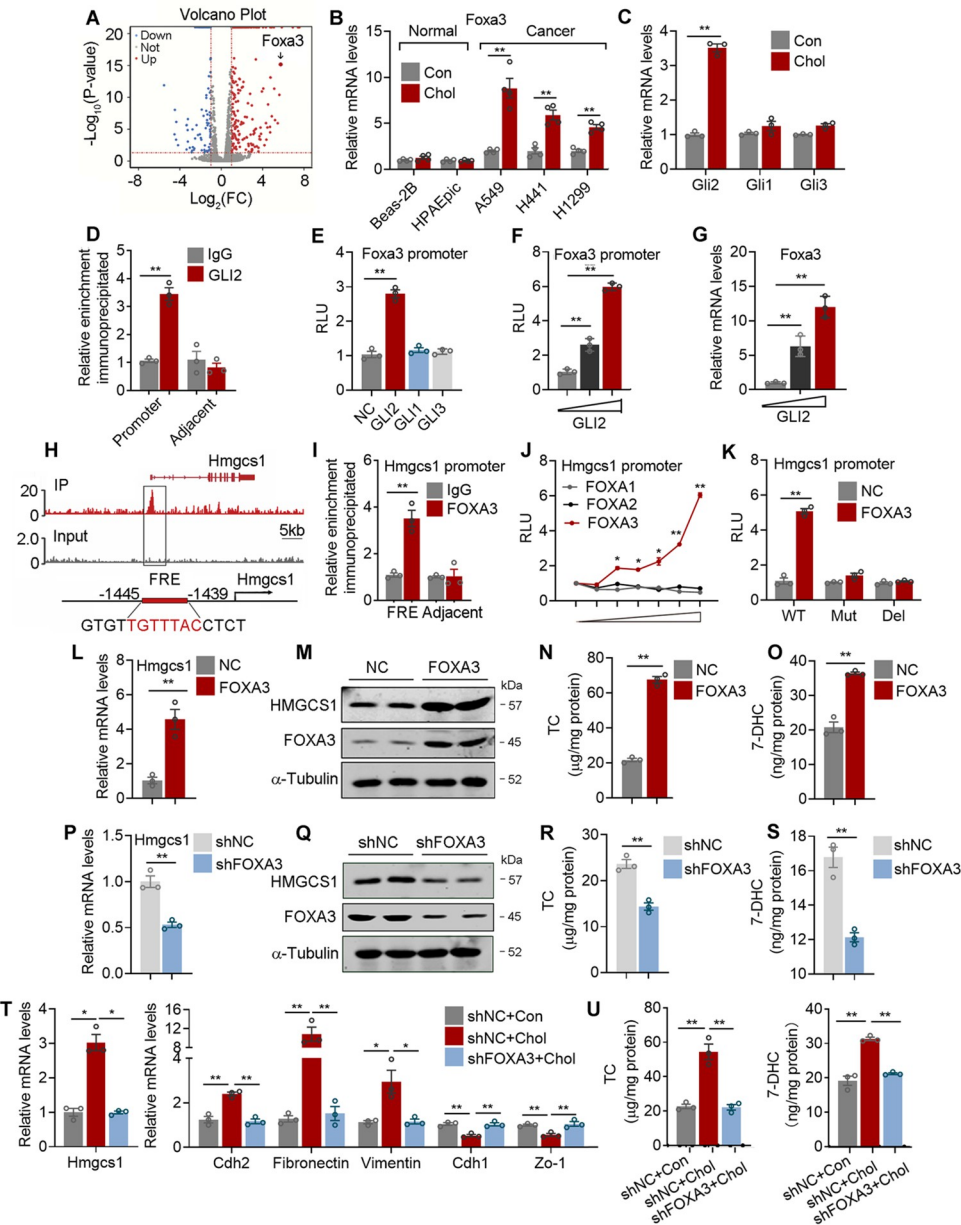
FOXA3 is a key transcriptional factor to mediate cholesterol-induced de novo cholesterol biosynthesis in lung adenocarcinoma cells. **(A)** Volcano analysis of differentially expressed genes of RNA-seq data of A549 cells treated with or without 5 μg/ml cholesterol (*p* < 0.05). Red, blue, and gray dots suggest up-regulation, down-regulation, and not changed, respectively. **(B)** Foxa3 mRNA levels of normal lung epithelia cells and lung adenocarcinoma cells treated with or without 5 μg/ml cholesterol for 24 h in LPDS medium (*n* = 4). **(C)** qPCR analysis of GLIs in A549 cells treated with or without 5 μg/ml cholesterol for 24 h in LPDS medium (*n* = 3). **(D)** Enrichment of GLI2 occupancy on Foxa3 promoter in A549 cells analyzed by ChIP assay (*n* = 3). **(E)** Effects of GLIs on Foxa3 transcriptional activities analyzed by luciferase assay (*n* = 3). **(F, G)** Dose-dependent effect of GLI2 on Foxa3 transcriptional activity analyzed by luciferase assay in 293T **(F)** and by qPCR analysis in A549 cells **(G)** (*n* = 3). **(H)** ChIP-seq visualization of FOXA3 occupancy on the proximal region (top) and in silico analysis of putative FRE (down) on Hmgcs1 promoter in H1299 cells. **(I)** The binding of FOXA3 on predicted FRE of Hmgcs1 promoter in A549 cells was analyzed by ChIP (*n* = 3). **(J)** Dose-dependent Hmgcs1 transcriptional activities in HEK293T cells transiently expressing either vector (PCDH) or FOXAs. **(K)** Luciferase activities of WT, FRE mutation (Mut), or FRE deletion (Del) Hmgcs1 promoter reporters in HEK293T cells transiently expressing either vector (NC) or FOXA3 (*n* = 3). **(L, M)** qRT-PCR **(L)** and immunoblotting **(M)** of FOXA3 and HMGCS1 levels in NC or FOXA3 overexpressed A549 cells (*n* = 3). **(N, O)** The cholesterol **(N)** and 7-DHC **(O)** levels in NC or FOXA3 overexpressed A549 cells (*n* = 3). **(P, Q)** qRT-PCR **(P)** and immunoblotting **(Q)** of FOXA3 and HMGCS1 levels in control (shNC) or FOXA3 knockdown (shFOXA3) A549 cells (*n* = 3). **(R, S)** The cholesterol **(R)** and 7-DHC **(S)** levels in control (shNC) or FOXA3 knockdown (shFOXA3) A549 cells (*n* = 3). **(T, U)** qRT-PCR analysis of Hmgcs1 and metastatic gene program **(T)**, cholesterol and 7-DHC **(U)** levels in in control (shNC) or FOXA3 knockdown (shFOXA3) A549 cells treated with or without cholesterol (*n* = 3). Data were presented as mean ± SEM. *, *P* < 0.05; **, *P* < 0.01. FC, fold change; RLU, relative luciferase unit; con, control; chol, cholesterol; IP, FOXA3 immunoprecipitation; NC, target gene overexpression control; FOXA3, forkhead box protein A3; FRE, FOXA3 response element; Del, FRE deletion; Mut, FRE mutation; shNC, target gene knockdown control; shFOXA3, FOXA3 knockdown; ChIP, chromatin immunoprecipitation. The data underlying this figure can be found in the Supporting information file S1 Data.

We next set out to study the mechanism that exogenous cholesterol activated de novo cholesterol synthesis. Dose and time-dependent transcriptional analysis revealed that cholesterol treatment significantly increased Hmgcs1 expression, without impacting critical factors in cholesterol uptake (Ldlr), classic cholesterol biosynthetic regulators (Srebp2, Insig, and Scap), rate-limiting enzyme in biosynthesis cascade (Hmgcr), or cholesterol efflux regulators (Lxr, Apoe, Abca1, Abcg1) in lung adenocarcinoma cells (S2A and S2B Fig). Besides, we did not detect USP20, a deubiquitinase recently reported to stabilize HMGCR in liver [33], in lung adenocarcinoma cells (S2A and S2B Fig). Interestingly, exogenous cholesterol treatment did not increase cholesterol synthesis in normal primary hepatocytes (S2C Fig), indicating these effects may be unique to lung cancer cells. Since extracellular signals often function by inducing changes in intracellular transcription factors, we then performed RNA-seq analysis (S2D Fig) on cholesterol-treated cells and control cells. GO and KEGG analysis revealed a significant enrichment of biological processes related to metastasis, including cell migration, cell junction assembly, and wound healing (S2E and S2F Fig). We also investigated key transcriptional factors that featured significant modulations during this process. Subsequent analysis rendered 34 differentially expressed transcriptional factors, among which FOXA3, a key transcription factor in lipid metabolism [21–25], was top ranked and piqued our interest (Figs 2A and S2G). Indeed, qPCR analysis confirmed that Foxa3 mRNA levels were significantly up-regulated upon cholesterol treatment in lung adenocarcinoma cells, but not in normal lung epithelial cells (Fig 2B). Exogenous cholesterol covalently modified and activated key factors in hedgehog signaling pathway to induce intracellular signaling cascade [6]. Consistently, we found increased level of Gli2, a downstream mediator of hedgehog signaling pathway, in our RNA-seq data (S2G Fig). This was further confirmed by a screen of GLIs family members in lung adenocarcinoma cells, which showed that cholesterol treatment specifically induced GLI2 expression (Fig 2C). Importantly, in silico analysis predicted a putative GLI2 binding site on Foxa3 promoter (S2H Fig), which was confirmed by ChIP assay (Fig 2D). Furthermore, GLI2, but not GLI1 or GLI3, enhanced luciferase activity of FOXA-luc construct (Fig 2E), while GLI2 overexpression dose-dependently promoted Foxa3 transcription in luciferase assay and in qPCR analysis (Fig 2F and 2G). These results suggested that exogenous cholesterol induced Foxa3 transcription in lung adenocarcinoma cells via GLI2.

Then, to elucidate the function of FOXA3 in de novo cholesterol synthesis, we performed ChIP-seq to examine its direct target genes, which revealed that HMGCS1 was top ranked (Fig 2H and S1 Table). Besides, overlapping RNA-seq dataset with ChIP-seq dataset revealed that Hmgcs1 is one of the top candidates that both served as direct target genes of FOXA3 and featured significant increase in expression levels upon cholesterol treatment (S2I Fig). HMGCS1 is the critical enzyme that governing the initial step in cholesterol biosynthesis, catalyzing energy substrates into HMG-CoA that is essential for mevalonate formation [34]. Consequently, FOXA3 overexpression or exogenous cholesterol treatment both increased the levels of HMG-CoA, the direct enzymatic product of HMGCS1 (S2J and S2K Fig). ChIP assay further confirmed FOXA3 binding at a canonical FOXA binding site from −1,445 bp to −1,439 bp on Hmgcs1 promoter (Fig 2H and 2I) [35]. In addition, FOXA3, but not its family member FOXA1 or FOXA2, dose-dependently promoted Hmgcs1 transcription in luciferase assay (Fig 2J), while deletion or mutation of the putative FOXA3 binding site on Hmgcs1 promoter abolished transcriptional activation of FOXA3 on Hmgcs1 (Fig 2K). Consistently, FOXA3 overexpression increased, while FOXA3 knockdown decreased Hmgcs1 expression, cellular cholesterol, and 7-DHC levels in lung cancer cells (Fig 2L–2S). Importantly, in accordance with our previous data, exogenous cholesterol treatment increased cellular cholesterol and 7-DHC levels, as well as enhanced the expressions of Foxa3, Hmgcs1, and metastatic gene program in lung adenocarcinoma cells, while these effects were abolished in the scenario of FOXA3 knockdown (Fig 2T and 2U). Overall, these data suggested that FOXA3 is a key mediator of exogenous cholesterol-induced de novo cholesterol biosynthesis and progression, possible via the GLI2-FOXA3-HMGCS1 regulatory axis, in lung adenocarcinoma.

### FOXA3 promotes lung cancer cell migration

**Fig 3:**
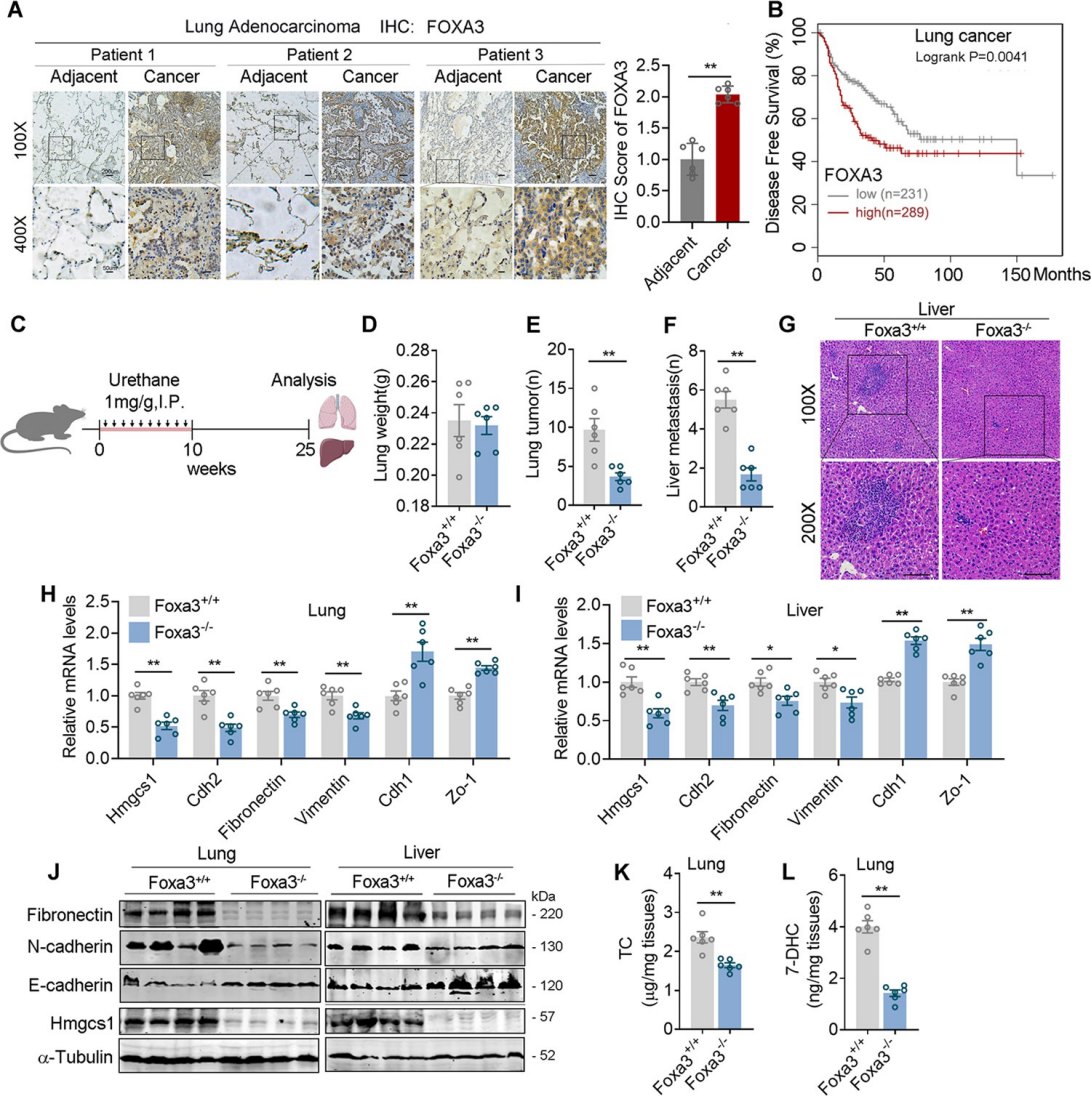
FOXA3 deletion alleviates lung cancer cell metastasis in genetic mouse model. **(A)** Representative images and quantification for FOXA3 nuclear staining of FOXA3 immunostaining in human lung adenocarcinoma samples and adjacent tissues (*n* = 6). **(B)** TCGA database of disease-free survival rate of lung adenocarcinoma patients with different Foxa3 expression levels (Foxa3 low, *n* = 231; Foxa3 high, *n* = 289). **(C–L)** Urethane-induced lung adenocarcinoma mice models **(C)**. Foxa3^+/+^ (*n* = 6) and Foxa3^-/-^ (*n* = 6) mice were intraperitoneally injected with 1 mg/g body weight urethane once a week for 10 weeks, and 15 weeks after last injection, mice were sacrificed and lung weights **(D)**, lung tumor numbers **(E)**, liver metastatic tumors **(F)**, and HE staining of liver tissues **(G)** were analyzed. **(H, I)** The expression of Hmgcs1 and metastatic gene program in lung **(H)** and liver **(I)** tissues of urethane-induced Foxa3^+/+^ and Foxa3^-/-^ mice. **(J)** The protein levels of Fibronectin, E-cadherin, N-cadherin, and HMGCS1 in lung (Left) and liver (right) tissues of urethane-induced Foxa3^+/+^ and Foxa3^-/-^ mice. **(K, L)** The cholesterol **(K)** and 7-DHC **(L)** levels in lung and liver tissues of urethane-induced Foxa3^+/+^ and Foxa3^-/-^ mice. Scale bars, 50 μm (G). Data were presented as mean ± SEM. *, *P* < 0.05; **, *P* < 0.01. IHC, immunohistochemistry; IP, intraperitoneal injection; Foxa3^+/+^, wild-type mice; Foxa3^-/-^, Foxa3 Knockout mice; TC, total cholesterol; 7-DHC, 7-dehydrocholesterol. The data underlying this figure can be found in the Supporting information file S1 Data.

Having shown that FOXA3 is involved in cholesterol synthesis, we next examined the contribution of FOXA3 to lung adenocarcinoma metastasis. We found in Oncomine database that lung adenocarcinoma tissues from patients characterized significantly higher FOXA3 expression compared to adjacent tissues (S3A Fig). We further confirmed enhanced FOXA3 levels in tumor tissues versus adjacent tissues using samples from lung adenocarcinoma patients by qPCR (S3B Fig), western blot (S3C Fig), and immunohistological analyses (Fig 3A). Moreover, Kaplan–Meier plotter for the correlation between FOXA3 levels and the disease-free survival of lung cancer patients from The Cancer Genome Atlas (TCGA) database revealed that high FOXA3 levels were associated with poor prognosis (Fig 3B).

To demonstrate the role of FOXA3 in lung adenocarcinoma metastasis, we investigated the influence of genetic FOXA3 deficiency in in vivo lung adenocarcinoma spontaneous metastasis models by treating WT (FOXA3^+/+^) and FOXA3 knockout (FOXA3^-/-^) mice with urethane, a chemical lung carcinogen (Fig 3C). We found that compared to WT mice, FOXA3^-/-^ mice had similar lung weight and significantly lower numbers of lung tumor nodes (Fig 3D and 3E). Of note, the migration of lung cancer to the liver was largely reduced in FOXA3^-/-^ mice (Fig 3F and 3G), accompanied with significantly reduced HMGCS1 and metastatic markers in both lung and liver, as well as suppressed cholesterol and 7-DHC levels in lung (Fig 3H–3L).

**Fig 4:**
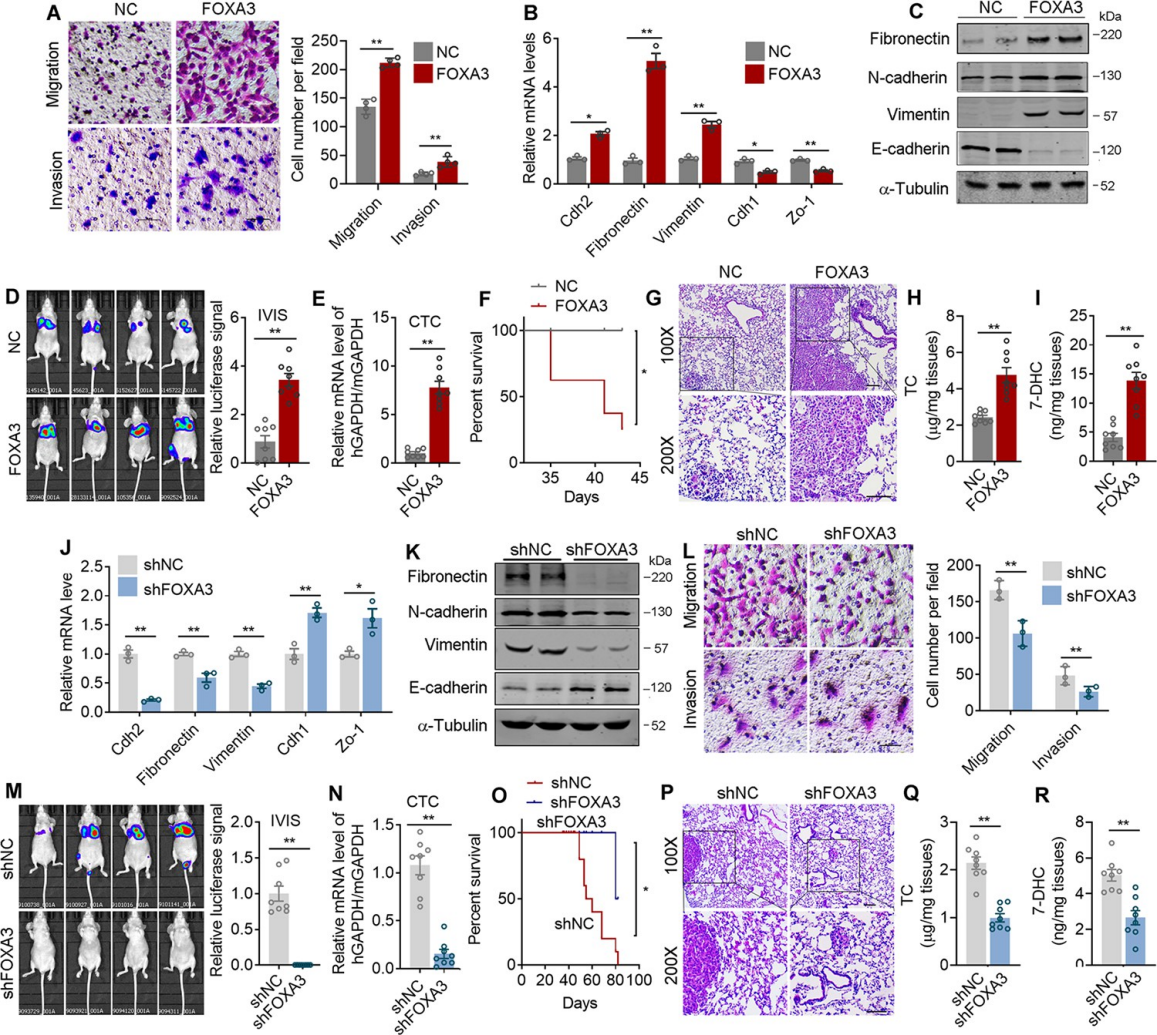
FOXA3 promotes lung cancer cell metastasis both in vitro and in vivo. **(A)** Representative images (left) and quantification (right) of transwell assay examining the effect of FOXA3 on A549 cell migration and invasion (*n* = 4). (**B, C)** The mRNA (*n* = 3) **(B)** and protein levels **(C)** of metastatic gene program of control (NC) and FOXA3 overexpressed (FOXA3) A549 cells. **(D–I)** Mice were injected intravenously with control (NC) or FOXA3 overexpressed (FOXA3) A549 cells (*n* = 8 per group). After 6 weeks, mice were analyzed for IVIS imaging **(D)**, qRT-PCR analysis of hGapdh/mGapdh in circulating tumor cells **(E)**, mice survival rate **(F)**, HE staining **(G)**, cholesterol levels **(H)** and 7-DHC levels **(I)** of lung tissues. **(J, K)** The mRNA (*n* = 3) **(J)** and protein levels **(K)** of metastatic genes in control (shNC) or Foxa3 knockdown (shFOXA3) A549 cells. **(L)** Representative images (left) and quantification (right) of transwell assay examining the effect of FOXA3 knockdown on A549 migration and invasion (*n* = 3). **(M–R)** Mice were injected intravenously with control (shNC) or FOXA3 knockdown (shFOXA3) A549 cells (*n* = 8 per group). After 8 weeks, mice were analyzed for IVIS imaging **(M)**, qRT-PCR analysis of hGapdh/mGapdh in CTC **(N)**, mice survival rate **(O)**, representative images of HE staining **(P)**, the cholesterol **(Q)** and 7-DHC levels in lung tissues **(R)**. Scale bars, 50 μm **(C, G, L, P)**. Data were presented as mean ± SEM. *, *P* < 0.05; **, *P* < 0.01. IVIS, In Vivo Imaging System; HE, hematoxylin and eosin staining; CTC, circulating tumor cells. The data underlying this figure can be found in the Supporting information file S1 Data.

Additionally, to eliminate the possibility that the suppressed tumor colonization to liver in FOXA3 ^-/-^ mice may be due to the reduced tumor incidence in their lung, we manipulated FOXA3 levels in lung adenocarcinoma cells and assessed its impacts on cancer migration ability both in vitro and in vivo. FOXA3 overexpression (FOXA3-OE) showed no effects on cell proliferation as shown by CCK8 analysis (S4A and S4B Fig), while significantly enhanced cell migration and invasion as shown by transwell assays (Fig 4A), expressions of metastatic gene program (Fig 4B and 4C), and wound-healing assays (S4C Fig). The impacts of FOXA3 on lung adenocarcinoma cell migration capability in vivo were further analyzed using xenograft mouse model. Mice were subjected to tail vein injection using luciferase-labeled FOXA3-OE lung cancer cells. Detailed analysis showed that compared to control group, FOXA3-OE cells resulted in stronger luciferase signal (Fig 4D), increased circulating tumor cells (CTC) in mice circulation (Fig 4E), reduced survival rate (Fig 4F), as well as increased lung colonization areas (Fig 4G), cholesterol (Fig 4H) and 7-DHC (Fig 4I) levels. Moreover, HMGCS1 levels and EMT markers (S4D Fig) in lung tissues were also elevated in FOXA3-OE xenograft mouse models. To further strengthen the role of FOXA3 in tumor metastasis, we have also established subcutaneous implantation models of murine lung cancer cells LLC with FOXA3 overexpression and examine the metastatic capability of these subcutaneous tumors compare to their controls. Consistently, FOXA3 overexpression promotes colonization of lung tissue and subsequent growth of subcutaneous LLC tumors (S4E and S4F Fig). It has been suggested that FOXA3 promoted metastasis of esophageal cancer cells through regulation of other FOXA members [36]. We thus examined FOXA1 and FOXA2 levels in A549 lung adenocarcinoma cells upon FOXA3 knockdown and found that the levels of FOXA1 and FOXA2 were not altered (S4G Fig), suggesting the regulatory role among FOXA family members might be cancer cell type specific. Conversely, FOXA3 knockdown (FOXA3-KD) cells exhibited opposite performance versus FOXA3-OE both in vitro and in vivo, as shown by similar proliferation rate (S4H Fig), decreased metastatic makers (Fig 4J and 4K), subdued in vitro cell migration/invasion (Figs 4L and S4I), as well as weaker luciferase signal (Fig 4M), less CTC in mice circulation (Fig 4N), increased survival rate (Fig 4O), decreased lung colonization areas (Fig 4P), decreased cholesterol (Fig 4Q) and 7-DHC levels (Fig 4R), as well as decreased HMGCS1 levels, along with the EMT markers (S4J Fig) in lung tissues of FOXA3-KD xenograft mouse models. We have also measured Srebp2 mRNA levels upon FOXA3 regulation in A549 and H441 lung cancer cells. The results showed that FOXA3 has no obvious effect on Srebp2 levels (S4K and S4L Fig). Overall, these data suggested that FOXA3 promotes lung cancer metastasis.

### HMGCS1 promotes lung cancer cell migration and is the downstream effector of FOXA3

**Fig 5:**
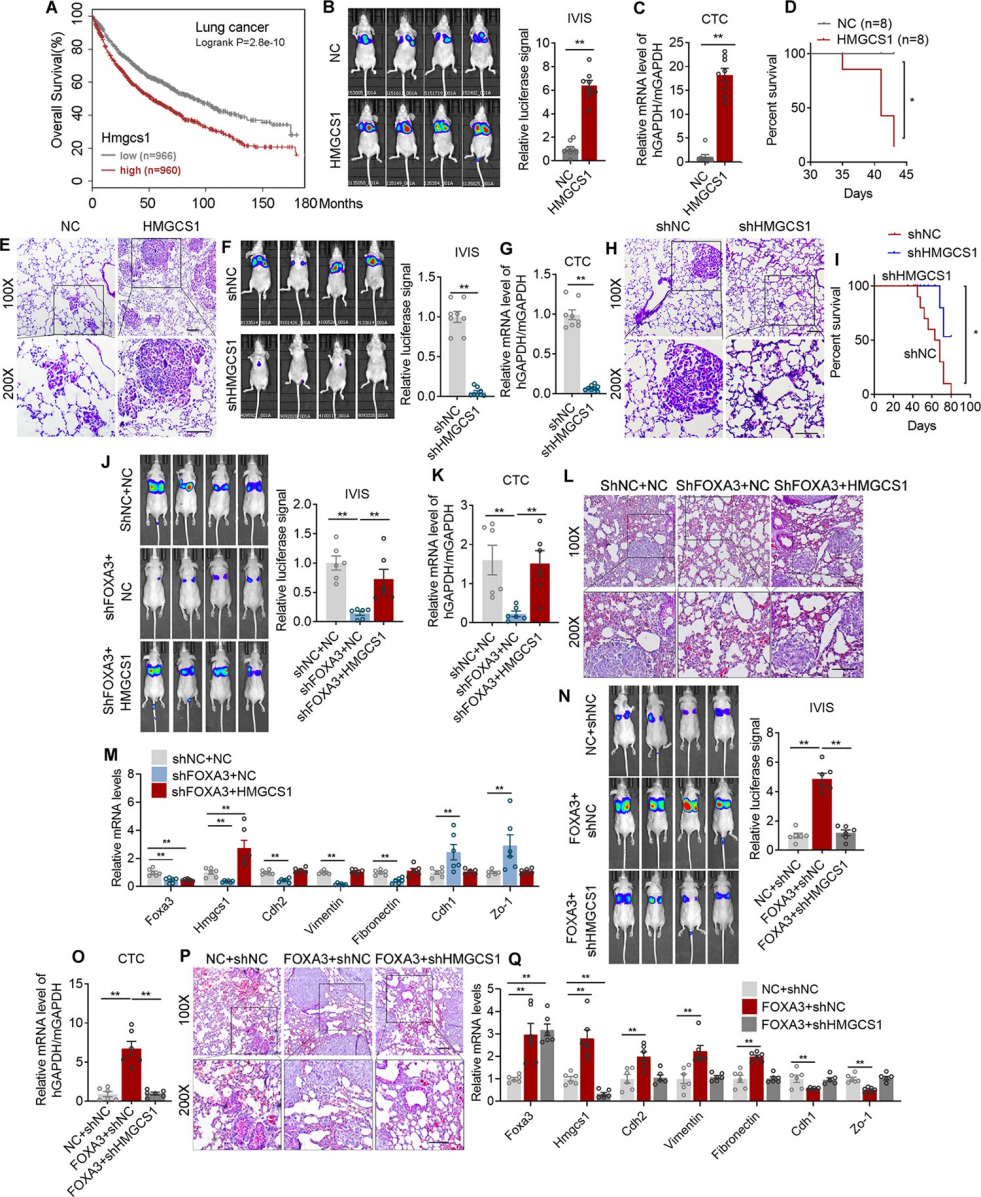
HMGCS1 promotes lung cancer cell metastasis both in vitro and in vivo and is the downstream effector of FOXA3. **(A)** Kaplan–Meier plotter showing the correlation between Hmgcs1 levels and the overall survival of lung adenocarcinoma cancer patients from TCGA database (Hmgcs1 low, *n* = 966; hmgcs1 high, *n* = 960). **(B–E)** Mice were injected intravenously with control (NC) or HMGCS1 overexpression (HMGCS1) A549 cells (*n* = 8 per group). After 6 weeks, mice were analyzed for IVIS imaging **(B)**, qRT-PCR analysis of hGapdh/mGapdh in circulating tumor cells **(C)**, mice survival rate **(D)**, and representative images of HE staining of lung metastatic nodules **(E)**. **(F–I)** Mice were injected intravenously with control (shNC) or HMGCS1 knockdown (shHMGCS1) A549 cells (*n* = 8 per group). After 8 weeks, mice were analyzed for IVIS imaging **(F)**, qRT-PCR analysis of hGapdh/mGapdh in circulating tumor cells **(G)**, representative images of HE staining of lung metastatic nodules **(H)**, and mice survival rate **(I)**. **(J–M)** Mice were injected intravenously with control (shNC+NC) or FOXA3 knockdown with (shFOXA3+HMGCS1) or without (shFOXA3+NC) HMGCS1 overexpression A549 cells (*n* = 6 per group). After 6–8 weeks, mice were analyzed for IVIS imaging **(J)**, qRT-PCR analysis of hGapdh/mGapdh in circulating tumor cells **(K)**, representative images of HE staining of lung metastatic nodules **(L)**, and the mRNA levels of metastatic gene program in lung tissues **(M)**. **(N–Q)** Mice were injected intravenously with control (NC+shNC) or FOXA3 overexpression with (FOXA3+shHMGCS1) or without (FOXA3+shNC) HMGCS1 knockdown A549 cells (*n* = 6 per group). After 6–8 weeks, mice were analyzed for IVIS imaging **(N)**, qRT-PCR analysis of hGapdh/mGapdh in circulating tumor cells **(O)**, representative images of HE staining of lung metastatic nodules **(P)**, and the mRNA levels of metastatic gene program in lung tissues **(Q)**. Scale bar, 50 μm **(E, H, L, P)**. Data were presented as mean ± SEM. *, *P* < 0.05; **, *P* < 0.01. IVIS, In Vivo Imaging System; HE, hematoxylin and eosin staining; CTC, circulating tumor cells. The data underlying this figure can be found in the Supporting information file S1 Data.

In addition to FOXA3, Kaplan–Meier plotter from TCGA database also revealed that high GLI2 and HMGCS1 levels were both positively correlated with poor prognosis and decreased overall survival in lung cancer patients (Figs 5A and S5A), suggesting the vital role of GLI2-FOXA3-HMGCS1 axis in lung cancer metastasis. Indeed, we found that HMGCS1 overexpression (HMGCS1-OE) in lung adenocarcinoma cells promoted lung cancer cell migration both in vitro (S5B and S5C Fig) and in vivo (Fig 5B–5E). Conversely, HMGCS1 knockdown (HMGCS1-KD) showed opposite effects (Figs 5F–5I, S5D and S5E). We have also established the subcutaneous implantation models of murine lung cancer cells LLC with HMGCS1 overexpression or knockdown. The analysis of metastatic capability of these subcutaneous tumors suggested that HMGCS1 overexpression promoted (S5F and S5G Fig), while HMGCS1 knockdown suppressed colonization of lung tissue and subsequent growth of subcutaneous tumors (S5H and S5I Fig).

Furthermore, we found that FOXA3 deletion inhibited lung colonization, while HMGCS1 overexpression rescued this phenomenon in mice (Fig 5J–5M). Moreover, FOXA3 overexpression promoted cancer cell migration both in vivo and in vitro, while HMGCS1 knockdown significantly abolished these effects (Figs 5N–5Q and S6A–S6D). These results suggested that HMGCS1 is the downstream effector of FOXA3 in cholesterol biosynthesis and lung adenocarcinoma progression.

### Cholesterol induces cell membrane composition change for lung adenocarcinoma progression

**Fig 6:**
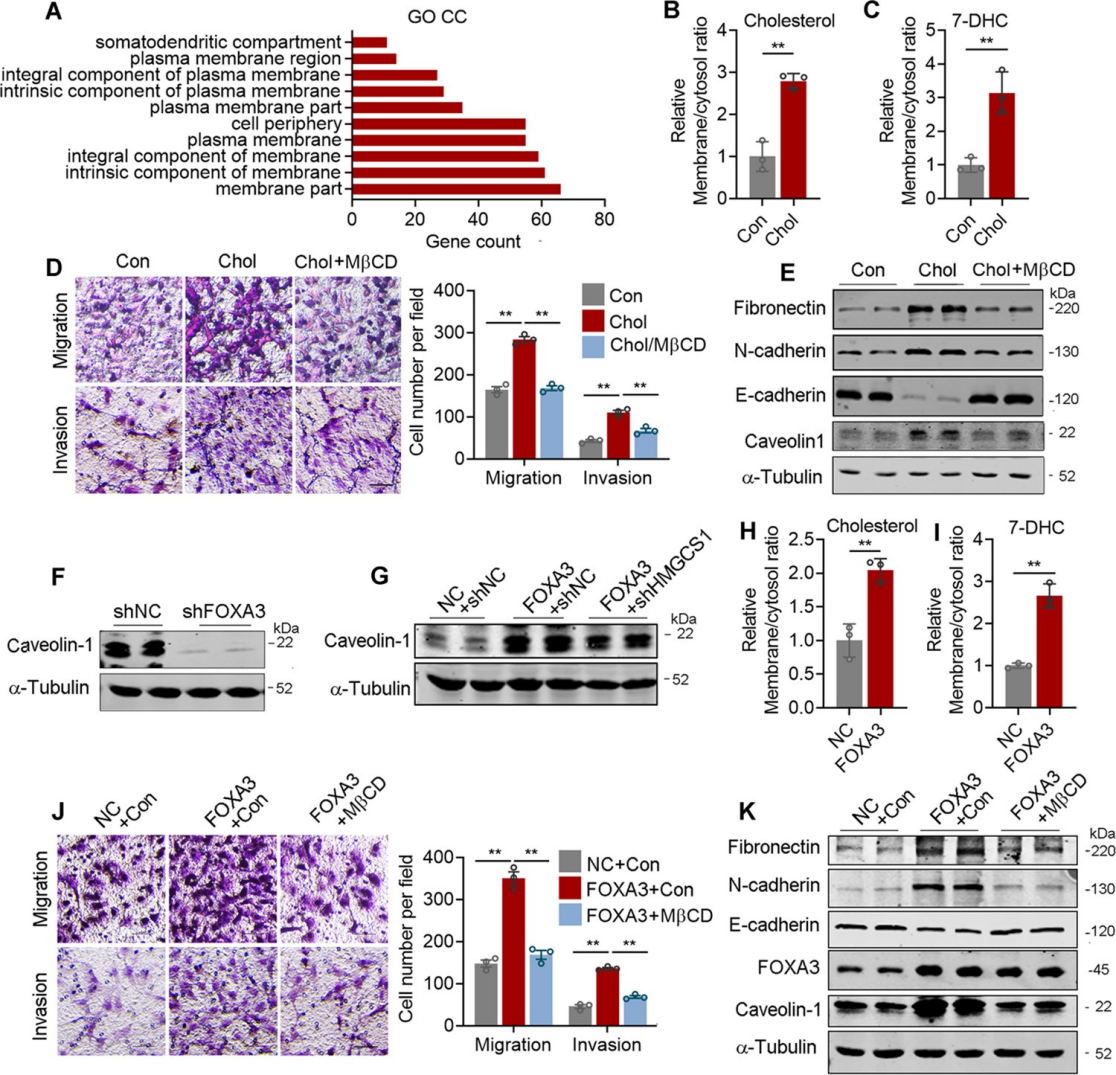
Cholesterol induces cell membrane composition change for lung adenocarcinoma cell migration. **(A)** GO enrichment analysis on differentially expressed genes (*p* < 0.05) from RNA-seq in A549 cells treated with or without 5 μg/ml cholesterol in LPSD medium for 24 h. **(B, C)** The ratio of cholesterol **(B)** and 7-DHC **(C)** levels in membrane or cytosol of A549 cells treated with or without 5 μg/ml cholesterol in LPSD medium for 24 h. **(D)** Representative images (left) and quantification (right) of transwell assay examining the migration or invasion of A549 cells treated with cholesterol (5 μg/ml) with or without MβCD (2.5 mM) pretreatment. **(E)** The effect of cholesterol with or without MβCD pretreatment on metastatic gene program and caveolin1 levels detected by immunoblotting. **(F, G)** The effects of FOXA3 knockdown **(F)**, FOXA3 overexpression with or without HMGCS1 knockdown **(G)** on the protein levels of caveolin-1 examined by immunoblotting. **(H, I)** The effect of FOXA3 on the ratio of cholesterol **(H)** and 7-DHC **(I)** levels in membrane or cytosol of A549 cells. **(J)** Representative images (left) and quantification (right) of transwell assay examining the migration or invasion of FOXA3 overexpressed A549 cells pretreated with or without MβCD for 24 h. **(K)** The effect of MβCD pretreatment on metastatic gene levels in FOXA3 overexpression A549 cells examined by immunoblotting. Scale bar, 50 μm **(D, J)**. Data were presented as mean ± SEM. *, *P* < 0.05; **, *P* < 0.01. *n* = 3 per group for cell experiments. GO, Gene Ontology; Con, Control; Chol, Cholestrol. The data underlying this figure can be found in the Supporting information file S1 Data.

We have shown that FOXA3 promoted de novo cholesterol synthesis and cell migration in lung adenocarcinoma via GLI2-FOXA3-HMGCS1 axis. In order to understand the influences of elevated intracellular cholesterol levels in lung adenocarcinoma cells, we analyzed RNA-seq data in Fig 2 with Gene Ontology (GO) analysis, which demonstrated that cellular membrane is top ranked and the most affected event after exogenous cholesterol treatment (Fig 6A), in accordance with previous report showing cholesterol as a critical contributor in membrane fluidity and membrane remodeling [37]. Indeed, we found that exogenous cholesterol treatment significantly increased cholesterol and 7-DHC incorporation into membrane compared to cytosol in lung adenocarcinoma cells (Fig 6B and 6C). Furthermore, exogenous cholesterol administration in lung adenocarcinoma cells resulted in enhanced cancer cell migration and invasion (Fig 6D), increased expression of metastatic markers, as well as Caveolin-1 (Fig 6E) and downstream signaling (S6E Fig). Notably, these effects were abolished by MβCD treatment, which disrupts membrane signal transduction (Figs 6D, 6E, and S6E).

We also found that FOXA3 levels in lung adenocarcinoma cells mimics the effects of exogenous cholesterol, as FOXA3-KD decreased caveolin-1 levels, while FOXA3-OE increased caveolin-1 levels and could be blocked by HMGCS1 knockdown (Fig 6F and 6G). Moreover, FOXA3-OE in lung cancer cells significantly increased cholesterol and 7-DHC incorporation into membrane versus cytosol (Fig 6H and 6I), as well as enhanced cell migration and metastatic marker expression, which were largely abolished by MβCD treatment (Fig 6J and 6K). Overall, these data suggested that membrane composition change at least partially contributed to FOXA3-HMGCS1 axis-induced cholesterol biosynthesis and metastasis in lung adenocarcinoma cell.

### Magnolol inhibits Foxa3 transcription and blocks metastatic program of patient-derived lung adenocarcinoma organoids

**Fig 7:**
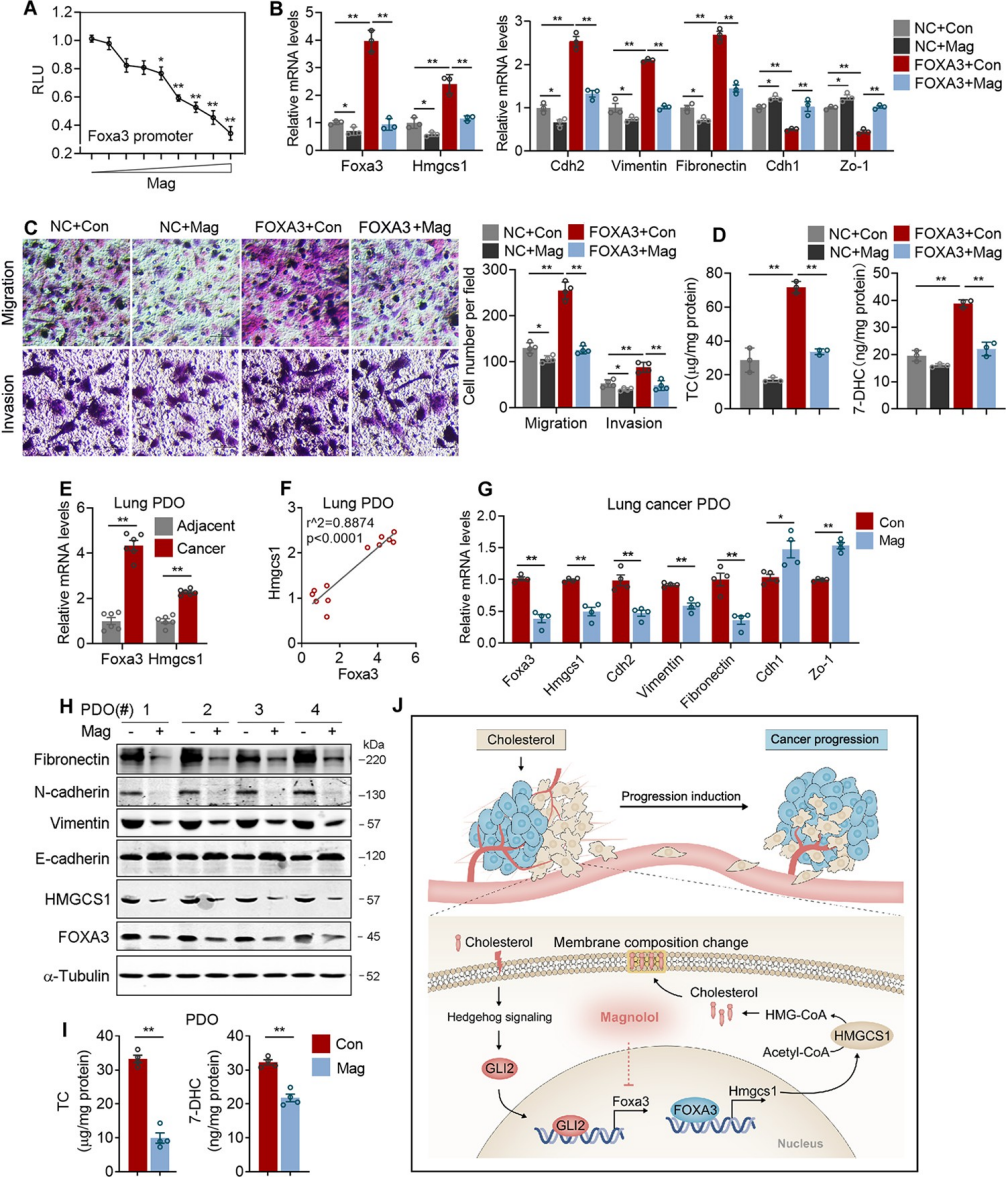
Magnolol as a lead compound against lung adenocarcinoma progression in PDOs. **(A)** Dose-dependent effect of magnolol on Foxa3 transcriptional activity. **(B)** qPCR analysis of the mRNA levels of Foxa3, Hmgcs1, and metastatic genesin FOXA3 overexpressed cells treated with or without magnolol at 10 μm (*n* = 3). **(C)** Representative images (left) and quantitation (right) of cell migration and invasion in FOXA3 overexpression A549 cells treated with or without magnolol (*n* = 4). **(D)** The cholesterol and 7-DHC levels of FOXA3 overexpressed A549 cells treated with or without magnolol (*n* = 3). **(E, F)** Relative mRNA levels of Foxa3 and Hmgcs1 **(E)** and correlation analysis **(F)** in lung adenocarcinoma PDOs (*n* = 6) and adjacent tissue PDOs (*n* = 6). **(G, H)** mRNA **(G)** and protein levels **(H)** of FOXA3, HMGCS1, and metastatic genes of lung adenocarcinoma PDO (*n* = 4) treated with or without 10 μm magnolol for 24 h. **(I)** The cholesterol (left) and 7-DHC (right) levels of lung adenocarcinoma PDO (*n* = 4) treated with or without magnolol. **(J)** Schematic illustration of exogenous cholesterol promotes endogenous de novo cholesterol synthesis via GLI2-FOXA3-HMGCS1 transcriptional axis, membrane composition change, and cancer metastasis in lung adenocarcinoma. Scale bar, 50 μm **(C)**. Data were presented as mean ± SEM. *, *P* < 0.05; **, *P* < 0.01. Mag, Magnolol; PDO, patient-derived organoid. The data underlying this figure can be found in the Supporting information file S1 Data.

Having established the role of FOXA3 in lung adenocarcinoma metastasis, we next examined whether pharmacological blockade of FOXA3 would lead to inhibition of metastasis. Via a Foxa3 transcriptional reporter screening (S2 Table), we identified magnolol, an active substance extracted from the bark of *Magnolia officinalis* and widely used as Chinese traditional medicine for its anti-inflammatory and antispastic effects, potently inhibits Foxa3 expression (Fig 7A). Of note, we demonstrated that magnolol treatment in lung adenocarcinoma cells effectively blocked FOXA3-OE-induced Hmgcs1 expression and metastatic gene program (Fig 7B), cell migration/invasion (Fig 7C), and de novo cholesterol synthesis (Fig 7D). These effects of magnolol are not due to toxicity towards lung adenocarcinoma cells, as the treating concentration does not affect cell viability (S7A–S7C Fig). In order to assess whether magnolol is a selective FOXA3 inhibitor, we investigated the effects of magnolol treatment on the expression of FOXA family members and the results suggested that magnolol suppresses FOXA3 expression specifically in a dose-dependent manner (S7D and S7E Fig). In addition, we analyzed the effects of magnolol on lung adenocarcinoma cells with or without FOXA3. Our results showed that magnolol treatment did not exert additional inhibitory effects on the expression of Hmgcs1 and EMT in the absence of FOXA3 (S7F and S7G Fig).

For clinical relevance, we obtained lung adenocarcinoma and adjacent tissue from patients to establish PDOs, a promising model for personalized cancer treatment. Consistent with our previous observation, Foxa3 and Hmgcs1 levels were higher in cancer PDO compared to adjacent PDO in lung adenocarcinoma patients (Fig 7E). Moreover, Hmgcs1 levels were positively correlated with Foxa3 levels in PDO samples (Fig 7F). In these PDO samples, we chose 4 samples featured highest Foxa3 and Hmgcs1 expression and subjected them for magnolol treatment. Importantly, magnolol effectively inhibited both mRNA (Fig 7G) and protein (Fig 7H) levels of metastatic gene program, as well as cholesterol and 7-DHC levels (Fig 7I) in these PDO samples, indicating magnolol as a promising lead compound in preventing lung adenocarcinoma development, especially in personalized treatment for cancers characterized high-Foxa3 and high-Hmgcs1 expression. In addition, we compared side-by-side the effects of magnolol (Mag) with other extensively studied anti-cancer therapies, i.e., fluvastatin (Fluv) and cisplatin (Cisp) in PDOs by examining cell viability (S7H Fig), proliferation marker Ki67 (S7I Fig), Hmgcs1 (S7J Fig), and EMT gene expressions (S7K–S7O Fig) to compare their anti-neoplastic and anti-metastatic effects on cancer cells. The results of in vitro PDO experiments showed that compared to fluvastatin and cisplatin, magnolol exhibited relatively mild effects on cell viability and proliferative genes, while strongly inhibited Hmgcs1 expression and cell metastatic gene programs. Furthermore, we established xenograft models using nude mice implanted with human A549 non-small-cell lung cancer (NSCLC) cells and treated them with magnolol or control to evaluate the effects of magnolol. The results demonstrated that magnolol exhibited significant inhibitory effects on lung adenocarcinoma colonization, suggesting its clinical translation potential (S8A–S8D Fig).

Taken together, our study suggested that lung adenocarcinoma cells responded to exogenous cholesterol with a unique positive feedback mechanism, which activated GLI2-FOXA3-HMGCS1 transcriptional axis to enhance de novo cholesterol biosynthesis and cellular cholesterol accretion, eventually promoted lung adenocarcinoma progression at least partially by promoting membrane composition change, while the FOXA3 inhibitor magnolol blocked this signaling cascade and prevented cancer metastasis (Fig 7J).

## Discussion

In this paper, we reported a previously unappreciated unique regulatory mechanism of cholesterol metabolism in lung adenocarcinoma, in which exogenous cholesterol functions as a signal molecular to activate de novo cholesterol biosynthesis via GLI2-FOXA3-HMGCS1 axis and promote cancer metastasis at least partially through enhanced membrane composition change. As an essential constituent of biomembranes and precursors for various hormones and lipid derivatives, cholesterol homeostasis is vital to numerous physiological systems, while its disturbance predisposes tumorigenesis. For example, cholesterol and its metabolites including bile acids and hormones, play complex roles in supporting cancer progression and suppressing immune responses [38]. Hypercholesterolemia has been shown to promote multiple types of cancer, including liver cancer, breast cancer, and prostate cancer, etc., whereas cholesterol is actively internalized and activate intracellular signaling by itself or via cholesterol metabolites to promote cancer development [10,39]. Intriguingly, previous studies investigated the roles of cholesterol in carcinogenesis were mostly focused on cholesterol-rich tissues like liver. Lung is typically characterized of low-cholesterol levels, where high-cholesterol level is even harmful to normal pulmonary functions [17]. However, previous lipidomic analysis indicated that lung adenocarcinoma, a cancer type with high metastatic capability, had high cholesterol content [19]. In this study, we demonstrated that in lung adenocarcinoma cells, exogenous cholesterol functions as a membrane signal to activate intracellular de novo cholesterol synthesis for membrane composition change. This unique cholesterol regulatory mechanism in lung adenocarcinoma could potentially bypass the limit of low-cholesterol uptake characterized of lung tissues and amplifies extracellular cholesterol signal to compensate for the incapability of uptake and usually low-cholesterol level in pneumonocytes. The newly synthesized cholesterol, along with intermediate metabolites of cholesterol biosynthetic pathway, i.e., 7-DHC, or cholesterol derivatives, i.e., 27-OH cholesterol [18], can in turn enhance lung adenocarcinoma metastasis via mechanisms likely including membrane composition change and signal transduction.

Our study highlighted a critical role of de novo cholesterol synthesis in cancer development in low-cholesterol tissues like lung, while this mechanism may be implicated in other types of cancers including HCC [40]. Indeed, although a causal relationship between high cholesterol and cancer development was established, disputes exist where low-cholesterol levels are sometimes associated with higher cancer incidents [41]. For example, Liang and colleagues reported that dietary cholesterol promotes hepatocellular carcinoma (HCC) through dysregulated metabolism and calcium signaling [42], while Wang’s group demonstrated that high serum cholesterol enhances Nature Killer Cells function and suppresses HCC [43]. Although previous reports indicated immune cell function is dependent on cholesterol levels for their anticancer ability, it is notable that our present study showing that tumor cells may utilize low serum cholesterol as a signal molecular to activate de novo cholesterol synthesis and promotes cancer development. Consistently, de novo cholesterol biosynthetic pathway, also known as the mevalonate (MVA) pathway [44], has been reported to be up-regulated in many cancer types [45,46], overall providing insights for the oncogenic mechanism of cholesterol in cancer progression, other than immune system activities.

Of note, in our study, we demonstrated that besides cellular cholesterol, de novo cholesterol synthesis produces 7-DHC, the precursor of cholesterol, which is then also incorporated into membrane to promote cancer metastasis. There exist 2 types of cholesterol precursor, 7-DHC and 24-DHC, which are converted into cholesterol by DHCR7 and DHCR24, respectively, as the last step of cholesterol biosynthesis [31]. 24-DHC has been shown to express in colorectal cancer [47], but the role of 7-DHC in cancer is lacking. Interestingly, though both 7-DHC and 24-DHC are cholesterol precursors, we found that in lung adenocarcinoma, DHCR7, the enzyme catalyzes 7-DHC into cholesterol, is dominantly expressed compared to DHCR24. Consistently, the activation of de novo cholesterol synthesis produced predominantly 7-DHC as its intermediate metabolite, with very low levels of 24-DHC. As cholesterol synthesis is a complexed anabolic metabolic pathway with numerous steps and many enzymes, it would be worthy to evaluate the unique spectrum of intermediate metabolites generated in this process and their specific functions in different types of cancers. Moreover, since high serum cholesterol levels favor cholesterol uptake and low serum cholesterol levels enhance de novo cholesterol synthesis, theoretically, intracellular cholesterol originated either from extracellular uptake or from intracellular synthesis should exert same function on cancer development, yet controversies exist concerning the correlation between serum cholesterol levels and tumor development [43,48,49]. The pro-tumor activity of 7-DHC we demonstrated in this study signifies a unique role of de novo cholesterol biosynthesis in carcinogenesis, which may potentially reconcile this controversy, as the various intermediate metabolite generated during cholesterol biosynthesis, including but not limited to 7-DHC, may have additional actions on tumor development, i.e., provide extra carbon chains direly needed by cancer cells for active anabolism and proliferation, or supply substrates for other metabolic pathways. Thus, the present study provides an extra layer to consider when evaluating the correlation between serum cholesterol levels and tumor development, especially in those tissue types capable of both active cholesterol uptake and biosynthesis.

We demonstrated that in lung adenocarcinoma, instead of being internalized, extracellular cholesterol binds to and transduces signals through cell membrane, which is blocked by reagents that interfere with membrane signal transduction. We further identified FOXA3 as the mediator of this extracellular cholesterol signaling. To date, the hedgehog signaling pathway is the only pathway regulated by extracellular cholesterol, during which cholesterol binds to and covalently modifies Hedgehog and smoothened (SMO), 2 key factors of the hedgehog pathway enriched on cell membrane [6], for downstream signaling activation [50]. Consistent with this, via RNA-seq and further molecular analysis, we found that cholesterol treatment enhanced the expression of GLI2, the downstream effector of SMO, and that GLI2 transcriptionally activated FOXA expression. FOXA3 is first identified as a key regulator of mature hepatocyte function [51], while we and others have demonstrated FOXA3 as a critical factor of energy hoarding that regulates lipid and glucose homeostasis in adipose tissues and in liver [21–23]. In the present study, we identified a vital role of FOXA3 as a mediator of exogenous cholesterol signaling and a target of the hedgehog signaling pathway, which transcriptionally activated HMGCS1 to promote de novo cholesterol synthesis, membrane composition change and metastasis in lung adenocarcinoma. The aberrant activation of hedgehog signaling pathway has long been demonstrated to be important to cancer development [52]. Though most studies focused on its pro-proliferating and pro-tumor growth roles, hedgehog pathway has also been shown to enhance cancer metastasis [53]. Our present study showed that FOXA3 is downstream of hedgehog pathway, which amplifies hedgehog signaling by promoting cholesterol synthesis and membrane composition change, thus mediates the metastatic function of hedgehog. Interestingly, compared to lung adenocarcinoma, FOXA3 levels are reported to be negatively correlated with HCC incidence [54]. In addition, previous reports have shown an important role of FOXA3 in maintaining normal hepatocytes functions, as co-administration of FOXA3 and other several factors can convert multiple types of cells, including hepatoma cells into hepatocyte-like cells [55,56]. Thus, future studies in the aspects of FOXA3 levels and cholesterol sensing and biosynthesis in different cancers may provide renewed angles for personalized treatment.

Cholesterol is de novo synthesized from acetyl-coenzyme A (acetyl-CoA) via more than 20 enzymes catalyzing complex reactions in the cholesterol biosynthetic pathway. HMGCR, the enzyme that converts HMG-CoA to MVA, is the rate-limiting enzyme of cholesterol synthesis [33]. The statin class of drugs, e.g., lovastatin, atorvastatin, and simvastatin, that targets HMGCR is effective in lowering cholesterol levels and is reported to exhibit pleiotropic antitumor effects [57]. However, chronic statins usage may lead to various side effects. For example, statins induced the compensatory increase in HMGCR levels due to the accumulation of upstream intermediate metabolites [58], which caused drug resistance. Moreover, myopathy, rhabdomyolysis, and liver injury, etc., are observed in statins treatment [59]. In this study, we demonstrated that Magnolol, a natural compound and an ingredient in Chinese medicine with long history, as a FOXA3 repressor. As the enzyme that catalyzes the first step of cholesterol synthesis, HMGCS1 converts acetyl-CoA and acetoacetyl-CoA into HMG-CoA, which then forms MVA by HMGCR [34]. Intriguingly, while the suppression of HMGCR renders an excessive accumulation of its upstream substrates and causes a feedback increase in HMGCR levels, HMGCS1 uses acetyl-CoA and acetoacetyl-CoA as its substrates, both of which are universal energy substrates that could be easily diverted and consumed by other metabolic pathways like the TCA cycle. Thus, HMGCS1 suppression by FOXA3 down-regulation via Magnolol may circumvent the dilemma seen in statin. Consistent with our study, Magnolol is reported to suppress cancer development in pancreatic cancer and colorectal cancer [60,61]. Though Magnolol has been shown to exert anti-cancer activity via inhibition of TGFβ signaling and NF-kβ signaling [61,62], it would be interesting to evaluate the roles of FOXA3-HMGCS1 axis in these cancers. In addition, inhibitors of SMO, the key factor in hedgehog signaling transduction, such as vismodegib, erismodegib, and glasdegib, have been used in clinic to treat cancers such as basal cell carcinoma, medulloblastomas, and acute myelogenous leukemia [63]. However, the effects of SMO inhibitors were sometimes invalidated due to the frequent SMO mutations in human patients [64,65]. Our study provided Magnolol as an alternative strategy, which may be especially relevant for cancer subtypes characterized of abnormal activation of hedgehog signaling and cholesterol biosynthesis.

In summary, our data revealed a positive feedback of cholesterol metabolism in lung adenocarcinoma and identified FOXA3 as a critical linker and regulator to sense cholesterol-hedgehog signaling and promote 7-DHC and cholesterol de novo biosynthesis via HMGCS1 for membrane composition change and progression. In addition, we also utilized magnolol as a lead compound to target FOXA3 and suppress cholesterol metabolism and potentially treat lung adenocarcinoma, overall providing insightful mechanism of cholesterol metabolism and potential targeting strategy against lung adenocarcinoma progression.

## Materials and methods

### Ethics statement

All animal studies were carried out following the guidelines approved by the Ethics Committee of Animal Experiments of East China Normal University (m20210254). Tumor and adjacent normal lung tissue were obtained and used for research purposes with approval from the Medical Ethical Council of Shanghai Chest Hospital. The written informed consent was obtained from all patients. The study abides by the Declaration of Helsinki principles.

### Cell culture and reagents

Beas-2B, HPAEpiC, A549, H441, H1299, HepG2, Huh7, and HEK293T cells were original purchased from the American Type Culture Collection (Manassas, Virginia, United States of America). LLC, HEK293T, HepG2, and Huh7 cells were cultured in DMEM, and Beas-2B, HPAEpiC, A549, H441, H1299 were cultured in RPMI 1640, supplemented with 10% FBS, 100 U/ml penicillin and 100 μg/ml streptomycin at 37°C in a humidified atmosphere with 5% CO2 except specially indicated. All cells were tested for mycoplasma contamination periodically. DMEM, RPMI1640, FBS, 100 U/ml penicillin and 100 μg/ml streptomycin, and 0.25% trypsin were purchased from Thermo Scientific (Gibco, Grand Island, New York, USA). Lentivirus system plasmids pCDH-CMV-MCS-EF1-Puro, pLKO.1-TRC, psPAX2, and pMD2.G were purchased from Addgene (Cambridge, Massachusetts, USA). Cisplatin (HY-17394), Fluvastatin (HY-14664), and Magnolol (HY-N0163) were purchased from MCE (Shanghai, China). Cholesterol (C3045), MβCD (C4555), and Filipin III (SAE0087) were purchased from Sigma (St. Louis, Missouri, USA). Human DiI-Low Density Lipoprotein (DiI-LDL) (20614ES76) was purchased from Yeason (Shanghai, China) and Cholesterol-biotin was purchased from Biobond (Biobond, Shanghai, China).

### Plasmids and shRNA lentiviral constructions

FLAG-tagged hFoxa3 and hHmgcs1 were generated by the ligation of the full-length open-reading frame into the pCDH-CMV-puro vector (Addgene, 167463). Myc-tagged GLI2, myc-tagged GLI1, and Myc-tagged GLI3 were generated by the ligation of the full-length open-reading frame into the pcDNA3.1 vector (Addgene, 182494). Plasmids for knockdown of Foxa3 or hmgcs1 were constructed into pLKO.1 vectors (Addgene, 10879), with a scrambled small hairpin RNA sequence (Addgene, 1864) serving as the control. The sequences for Foxa3 or Hmgcs1 knockdown are listed in S3 Table. psPAX2 (12260, Addgene) and pMD2.G (12259, Addgene) were used to prepared lentivirus. High-titer lentivirus was packaged in HEK293T cells using EZ Trans (Life iLab, Shanghai, China). Viral supernatants were collected 72 h after transfection, and the lentivirus was concentrated by ultracentrifugation, 0.45 μm filtered and applied to cancer cells in the presence of 1 μg/ml polybrene for 24 h. Cells were selected with 1 μg/ml puromycin for 10 days. Gene expression levels were confirmed by qPCR and immunoblotting.

### Mice and tumor models

All mice were maintained under a standard humidity- and temperature-controlled environment on a 12-h light/dark cycle, with free access to food and water. All mice in our experiments were gender and age matched. For lung metastasis models, 6-week-old male BalB/C nude mice were purchased from Shanghai Research Center for Model organisms, and 5 × 10^6^ luciferase-labeled A549 cells were injected into mice via tail vein. The tumors were detected by luciferase-based noninvasive bioluminescence imaging using the platform of IVIS Lumina II (PerkinElmer, Massachusetts, USA), and 6 to 8 weeks later, mice were euthanized and lung metastasis were measured by counting lung surface tumor number and by HE staining of lung paraffin sections. For magnolol treatment, magnolol (15 mg/kg) was administrated subcutaneously every other day for about 6 weeks. Wild-type (WT, Foxa3^+/+^) and FOXA3-null (Foxa3^-/-^) mice have been previously described [23]. For urethane-induced lung cancer, 8-week-old male Foxa3^+/+^ and Foxa3^-/-^ mice were injected intraperitoneally with 1 g/kg urethane (ethyl carbamate; Sigma, Missouri, USA) 1 injection per week for 10 weeks. Mice were monitored once per week, and the lung and liver tissues of mice were collected 15 weeks after urethane treatment. The number of lung tumor bearing mice in each group was observed and counted and the tumor size was recorded by the diameter.

For LLC spontaneous lung metastatic models, to initiate the study, 1 × 106 LLC cells with FOXA3 overexpression, HMGCS1 overexpression, or HMGCS1 knockdown were suspended in 200 μl of phosphate-buffered saline (PBS) and subcutaneously injected into the right flank of mice. Upon reaching a size of 100 mm^2^ (about 3 weeks), the tumors were surgically excised. Subsequently, 20 to 26 days post-tumor removal, lung metastasis was evaluated through HE staining on paraffin sections of lung tissue.

### RNA extraction and real-time PCR

Total RNA was extracted using RNAiso Plus (Takara, Beijing, China) according to the manufacturer’s instructions. The mRNA was reverse transcribed using PrimeScript RT Master Mix (Takara, Beijing, China). Real-time PCR was performed using SYBR Green qPCR Master Mix with Roche 480 system. The relative expression levels were measured using the relative quantitation ΔΔCt method and normalized to gapdh expression. The primers used for real-time PCR are listed in S3 Table.

### Immunoblotting

Cell lysates were prepared and immunoblotings were performed as previously described [66]. Briefly, tissues were homogenated and cells were lysed in RIPA lysis buffer. Protein samples were electrophoresed on SDS-PAGE and transferred onto nitrocellulose membranes. The membranes were incubated with appropriate primary antibodies overnight at 4°C, washed with TBST and incubated with IRDye secondary antibodies (926–68071 and 926–322210) from LI-COR Biosciences (LI-COR biosciences, Nebraska, USA). The protein signals were detected by Odyssey CLX imaging system (LI-COR biosciences, Nebraska, USA). α-tubulin was used as a loading control. Antibodies used for immunoblotting are listed in S4 Table.

### Immunohistochemistry

Dehydrated paraffin sections were pretreated with 3% H2O2 for 30 min to quench endogenous peroxidase activity and then blocked in PBS containing 2.5% horse serum. FOXA3 staining were performed by incubating with mouse anti-FOXA3 (sc-74424, Santa Cruz) in a humidified chamber at 4°C overnight. Sections were washed with PBS and incubated with IgG-HRP (Huabio, Hangzhou, China) secondary antibody at room temperature. The signal was detected by DAB kit (Servicebio, Wuhan, China). Images were acquired using an Olympus microscope and quantitative analysis was conducted with ImageJ.

### Immunofluorescence

A549 or HepG2 were cultured on coverslips in 24-well plates for 24 h, fasted for 12 h, and treated with or without 2.5 mM MβCD for 30 min, cells were washed thoroughly with RPMI1640 medium twice, then switched to fresh RPMI1640 medium supplemented with 5% lipoprotein-deficient serum (LPDS medium) [66] and treated with 5 μg/ml Cholesterol-Biotin for 24 h. Cells were then fixed, permeabilized, and incubated with primary antibodies against biotin (Abclonal, A18848) overnight at 4°C. Cells were then washed and incubated with Cy3 Goat Anti-Rabbit (Abclonal, AB_2769089). Dil-LDL uptake was performed as previously described [67]. Briefly, the cells were put in LPDS medium at 4°C for 30 min, then switched to fresh LPDS medium supplemented with 20 μg/ml Dil-LDL at 4°C for 1 h. Cells were then incubated at 37°C with fresh LPDS medium and fixed with 4% paraformaldehyde for visualization. Filipin III (Sigma, SAE0087) staining was performed according to manufacturer’s protocol. Briefly, cells were fixed with 4% paraformaldehyde (PFA) and stained with 50 μg/ml filipin III for 30 min at 4°C for visualization. DAPI was used to visualize the nuclei. Images were collected using a Leica SP8 confocal microscope and analyzed using a Leica LAS AF software. Quantitative analysis was conducted with ImageJ.

### Cell migration and invasion

The assay was performed using Transwell migration chambers (8 mm pore size; Corning Costar, New York, USA) according to the instructions as previously described [67]. For invasion assays, the chambers were precoated with matrigel (356231, Corning, New York, USA) according to the instructions. For both assays, 1 × 10^5^ cells were suspended with serum-free culture medium and seeded into the upper well of the chamber, the lower well was filled with normal culture medium containing 10% FBS functions as a chemoattractant. Cells were cultured for 16 h in migration assay and 24 h in invasion assay, the non-migrated or noninvasive cells were scratched from the upper surface of the transwell. Cells on the lower surface of the Insert chambers were fixed with neutral formalin, stained with hemotoxylin/eosin, and counted under Nikon digital camera (Tokyo, Japan) at ×200 magnification.

### Detection of circulating tumor cells

The relative circulating tumor cells (CTCs) was examined by detecting the ratio of hGapdh expression to mGapdh expression by real-time PCR. The primers were listed in S3 Table.

### Total cholesterol, dehydrocholesterol, and HMG-CoA detection

For cellular assays evaluating intracellular cholesterol, dehydrocholesterol, and HMG-CoA levels, cells were cultured in LPDS medium (culture medium plus 5% LPDS) before indicated manipulation. Cholesterol levels were examined with a total cholesterol assay kit (Sigma), and 7-dehydrocholesterol, 24-dehydrocholesterol, and HMG-CoA levels were detected with ELISA kits (Zeye Bio and Milbio, Shanghai, China) following the manufacturer’s instructions.

### Drug screening and luciferase reporter assays

A collection of 850 Natural compounds was obtained from targetmol (Wellesley Hills, Massachusetts, USA). Foxa3 or control reporter plasmids with pRL-SV40 (Renilla luciferase control reporter vector) were co-transfected into HEK293T cells by lipofectamine 2000 (Invitrogen, New York, USA). Cells were plated into 96-well plates and were added each of 850 Natural compounds at 10 μm. Luciferase activity was measured using the Dual-Luciferase Reporter Assay System (Promega).

### Lung cancer clinical specimens and PDO

PDOs were established by BioGenous as previously reported [68]. Briefly, tissue derived from surgical resections was cut into small pieces and enzymatically digested using 800 U/ml collagenase I (GIBCO) and 10 mM Y-27632 (Sigma-Aldrich) before embedding in Matrigel (Corning). After Matrigel solidification for 15 min at 37°C, cells were overlaid with human normal airway or NSCLC organoid medium. Human normal airway and NSCLC organoid medium is composed of 1× Advanced DMEM/F12 (GIBCO), 1× Glutamax (GIBCO), 100/100 U/ml Penicillin/Streptomycin (GIBCO), 10 mM HEPES (GIBCO), 1× B27 supplement (GIBCO), 1.25 mM N-Acetylcystein (Sigma-Aldrich), 10 mM nicotinamide (Sigma-Aldrich), 500 ng/ml human recombinant R-spondin1 (OrganRegen), 100 ng/ml human recombinant Noggin (OrganRegen), 25 ng/ml human recombinant FGF-7 (OrganRegen), 100 ng/ml human recombinant FGF-10 (OrganRegen), 500 nM A83-01 (Selleck), 500 nM SB202190 (Selleck), 5 mM Y-27632 (Selleck), and 1× Puromycin (Invitrogen) was added to prevent microbial contamination. Organoids were passaged approximately every week by incubating in TrypLE Express (GIBCO) for 2 to 5 min at 37°C to dissociate organoids and replating in fresh Matrigel. Organoids were cryopreserved in Organoid Cryopreservation Medium (bioGenous) as master and working biobanks. Organoids < passage 20 were used in experiments.

### RNA-seq preparation and RNA-seq data analysis

RNA-seq preparation and RNA-seq data analysis were performed as previously reported [69]. Briefly, total RNA was isolated using the standard RNAiso Plus Protocol and RNA quality was examined. RNA samples were purified with magnetic oligo(dT) beads after denaturation and were reverse transcribed into first-strand cDNA. Fragmented DNA samples were end blunted and adenylated at the 3′-end. Adaptors were ligated to construct a library. After DNA quantified by Qubit (Invitrogen, New York, USA) and cBot cluster generation, DNA samples were then sequenced by an Illumina HiSeqX Ten SBS instrument (Genergy Bio, Shanghai, China). Raw data were converted into Fastq format. The number of transcripts of each sample was calculated based on the number of fragments per kilobase of transcript per million fragments mapped (FPKM). The FPKM value was calculated by Cuffnorm software for each sample, and the values were log2 transformed. The differential gene transcripts between different samples were calculated using DESeq software. The thresholds for determining differentially expression transcripts are *P* < 0.05 and fold change≥2. Processed RNA-seq data were filtered by removing genes with low read counts. Read counts were normalized using TMM normalization and CPM (counts per million) were calculated to create a matrix of normalized expression values. Selected gene sets were annotated by GO analysis by DAVID database (DAVID 6.8) [66]. Heatmap was generated using heatmapper website (http://www.heatmapper.ca/expression/). The raw data of RNA-seq were uploaded into the NCBI Sequencing Read Archive (SRA) under accession number PRJNA845911.

### Membrane and cytosol components extraction

A549 cells were treated with 5 μg/ml cholesterol in RPMI1640 containing 5% LPDS medium for 24 h, the cytosol and membrane components were obtained using Membrane and Cytosol Protein Extraction Kit (Beyotime, Shanghai, China) according to the manufacturer’s protocol and stored at −80°C for further analysis.

### LC-MS analyses for cholesterol and its precursors

^13^C-acetate was used as a tracer to evaluate the impact of exogeneous cholesterol on de novo cholesterol synthesis. A549 cells were maintained in RPMI1640 containing 5% LPDS (Invitrogen). Prior to incubation with cholesterol or ^13^C tracers, A549 cells were fasted for 16 h, then A549 cells were washed with PBS and incubated with RPMI1640 containing 5% LPDS (Invitrogen) and 5 mM ^13^C-acetate (Sigma), and cells were treated with or without cholesterol for 24 h. Then, the cells were collected and stored at −80°C for further analysis.

Cholesterol and its precursors, dehydrocholesterol in cells or cellular components were extracted with chloroform/methanol mix solution and analyzed using an I-Class UHPLC coupled to a Q-TOF mass spectrometer (Waters Corp., Milford, Massachusetts, USA) under positive ESI mode. A Q-TOF mass analyzer was operated at 22,000 mass resolution with the scan range from 50 to 1,500 m/z. The data were analyzed by MassLynx v4.2 software (Waters Corp., Milford, Massachusetts, USA) and normalized with internal standard and protein concentration measured by the BCA protein assay kit.

### ChIP and ChIP-seq

ChIP was performed using a SimpleChIP Plus Enzymatic Chromatin IP Kit (9003, Cell signaling) according to manufacturer’s instructions. Briefly, Flag-tagged FOXA3 overexpressed H1299 lung cancer cells were cross-linked by 1% formaldehyde for 10 min and stopped by 0.125 M glycine solution. Cells were then washed twice with ice-cold PBS and scraped into PBS, the extracted chromatin was digested and fragmented into 200 to 1,000 bp by the Bioruptor (Scientz–IID; Scientz) in SDS lysis buffer, and then immunoprecipitated by ChIP grade antibodies against Flag (F1804, Sigma) or a normal IgG (12–371, Sigma) using Magnetic Beads. After that, the DNA was uncross-linked with proteins. PCR was then performed using the primers of the putative FOXA3 binding site. Primers were listed in S3 Table.

For ChIP-seq assay, a total amount of 50 ng DNA per sample was used to generate libraries using the NEBNext Ultra II Library Prep Kit for Illumina (NEB), reactions were prepared according to the manufacturer’s manual. Libraries were sequenced 50 bp single-end on an Illumina HiSeq2500 platform (Illumina). Alignments were filtered by Skewer to exclude reads with low quality, adapter and short (less than 50 bp) sequence fragments. For data analysis, Fastqc software was used to carry out the quality of the processed sequences. Then, the filtered sequencing reads were mapped to the human reference sequence for the human genome using Bowtie2 (v.2.2.5), the original sequence of the sample was compared with the reference genome for unique mapping analysis and filtered the sequences that can align to multiple (>1) genome locations. After unique alignment, duplicated reads were removed.

For peak calling, MACS2 call peak (v2.1.1) was used for the enrichment regions of reads on the genome for each ChIP replicates and Input as background separately, using *p* value <0.01. The raw data of ChIP-seq were uploaded into the NCBI SRA under accession number PRJNA845827. The regarding binding genes were listed in S1 Table.

### Cell viability

For cell lines, the assay was conducted using an Enhanced Cell Counting Kit-8 (CCK8, C0042, Beyotime) following the manufacturer’s instructions. Briefly, 5,000 cells per well in 100 μl were seeded in 96-well plates. After 24 h, the cells were treated with the magnolol at the indicated concentrations for an additional 24 h. Subsequently, CCK8 were added to each well and incubated for 0.5 to 2 h for further analysis.

For PDO cell viability assessment, we employed the ATP cell viability assay (Biogenous E238003) following the manuscript instructions. Briefly, PDO was added with an equivalent volume of the assay reagent to the culture plate, following with vigorously shake for 5 min and incubation at room temperature for 20 min. Analysis of the relative vitality of the organoids was based on the obtained chemiluminescence reading at a wavelength of 560 nm.

### Online data mining

Clinical data of Hmgcs1 and Gli2 expression analysis for lung patients were downloaded from the Kaplan–Meier Plotter (https://kmplot.com/analysis). Clinical data of Foxa3 expression analysis for lung patients were downloaded from GEPIA (http://gepia.cancer-pku.cn/) [70] or Oncomine (https://www.oncomine.org/). In the analysis process, gene expression data and relapse free and overall survival information are downloaded from TCGA and the GTEx projects, using a standard processing pipeline. The database is handled by a PostgreSQL server, and gene expression and clinical data were integrated simultaneously. The survival plot and logrank *P* value are calculated and plotted.

### Statistical analysis

Data analyses were performed by Graph-Pad Prism 8 (GraphPad Software). Unpaired Student’s *t* test was used for comparison between 2 groups. Log-rank (Mantel–Cox) test was used to analyze survival curve. The data are shown as means ± SEM. *p* < 0.05 was considered as statistically significant, *p < 0.05, **p < 0.01.

## Supporting information

S1 Fig Related to Fig 1. **(A)** The cholesterol levels in lung, breast, brain, and liver tissues of normal chow diet-fed mice (*n* = 5). **(B, C)** The mRNA (*n* = 3) **(B)** and protein **(C)** levels of metastatic genes in A549 cells cultured in LPDS medium and treated with 5 μg/ml cholesterol for 24 h. **(D)** Representative images (left) and quantification (right) of transwell assay examining the effect of exogenous cholesterol on cell migration of A549 cells (*n* = 3). **(E, F)** The effects of cholesterol on lung cancer cells viability. **(F, G)** Quantitative analysis of Fig 1E–1G were conducted using Image J (*n* = 4). **(H)** The cholesterol levels of A549 cells treated with 5 μg/ml cholesterol and/or MβCD for 24 h (*n* = 6). **(I)** The intracellular cholesterol levels detected by Filipin III in A549 cells treated with 5 μg/ml cholesterol for 24 h with or without MβCD pretreatment. Scale bar, 50 μm **(D, I)**. Data were presented as mean ± SEM. *, *P* < 0.05; **, *P* < 0.01. Con, control; Chol, Cholesterol; Memb, Membrane; Cyto, Cytosol. The data underlying this figure can be found in the Supporting information file S1 Data.
(TIF)

S2 Fig Related to Fig 2. **(A, B)** Dose and time-dependent qPCR analysis of the mRNA levels of key cholesterol metabolism-related genes in cholesterol-treated A549 cells. **(C)** qPCR analysis of the mRNA levels of key cholesterol synthesis genes in primary hepatocytes (*n* = 6). **(D–G)** PCA of genes **(D),** GO-BP **(E)**, and KEGG **(F)** and heat map of main transcriptional factors **(G)** of RNA-seq of A549 cells treated with or without cholesterol. **(H)** In silico analysis of putative GLI2 binding site on FOXA3 promoter. **(I)** Venn graph of the DEGs of RNA-seq and enriched genes of ChIP-seq. **(J)** The HMG-CoA levels in control (NC) or FOXA3 overexpressed A549 (left) or H441 (right) cells (*n* = 3). **(K)** The HMG-CoA levels in A549 (left) or H441 (right) cells treated with or without 5 μg/ml cholesterol in LPDS medium (*n* = 3). Data were presented as mean ± SEM. *, *P* < 0.05; **, *P* < 0.01. Con, control; Chol, Cholesterol; principal component analysis; DEG, differentially expressed genes. The data underlying this figure can be found in the Supporting information file S1 Data.
(TIF)

S3 Fig Related to Fig 3. **(A)** Scatter diagram of Foxa3 levels in lung adenocarcinoma and adjacent tissues from oncomine database. **(B, C)** Foxa3 mRNA **(B)** and protein **(C)** levels in lung adenocarcinoma and adjacent tissues (*n* = 10 per group). Data were presented as mean ± SEM. *, *P* < 0.05; **, *P* < 0.01. Ad, Adjacent tissues; Ca, lung cancer tissues. The data underlying this figure can be found in the Supporting information file S1 Data.
(TIF)

S4 Fig Related to Fig 4. **(A, B)** Foxa3 mRNA levels (*n* = 3) **(A)** and cell viability (*n* = 6) **(B)** in control (NC) or Foxa3 overexpressed A549 cells. **(C)** Representative images (left) and quantification (right) of wound healing assay examining the effect of FOXA3 overexpression on A549 cell migration. **(D)** Immunoblotting analysis of HMGCS1 and EMT-related proteins in lung tissues of nude mice injected with FOXA3-overexpressed A549 cells. **(E, F)** Subcutaneous injection of FOXA3-overexpressed LLC in a lung cancer metastasis model, HE staining **(E)**, mRNA expression analysis **(F)** of lung tissues (*n* = 6). **(G, H)** FOXA family members mRNA levels (*n* = 3) **(G)** and cell viability (*n* = 6) **(H)** in control (shNC) or Foxa3 knockdown (shFOXA3) A549 cells. **(I)** Representative images (left) and quantification (right) of wound healing assay examining the effect of FOXA3 knockdown on A549 cell migration. **(J)** Immunoblotting analysis of HMGCS1 and EMT-related proteins in lung tissues of nude mice injected with FOXA3-knockdowned A549 cells. **(K, L)** Srebp2 mRNA levels of FOXA3-overexpressed and FOXA3-knockdowned A549 **(K)** and H441 **(L)** cells (*n* = 3). Scale bar, 200 μm **(C, I)**, scale bar, 50 μm **(E)**. Data were presented as mean ± SEM. *, *P* < 0.05; **, *P* < 0.01. The data underlying this figure can be found in the Supporting information file S1 Data.
(TIF)

S5 Fig Related to Fig 5. **(A)** Kaplan–Meier plotter showing the correlation between GLI2 levels and the overall survival of lung adenocarcinoma cancer patients from TCGA database (GLI2 low, *n* = 964; GLI2 high, *n* = 961). **(B)** qRT-PCR analysis of metastatic gene program in control (NC) or HMGCS1 overexpressed (HMGCS1) A549 cells (*n* = 3). **(C)** Representative images (left) and quantification (right) of transwell assay examining the effect of HMGCS1 overexpression on A549 cell migration and invasion (*n* = 4). **(D)** qRT-PCR analysis of metastatic gene program of control (shNC) and HMGCS1 knockdown (shHMGCS1) A549 cells (*n* = 3). **(E)** Representative images (left) and quantification (right) of transwell assay examining the effect of HMGCS1 knockdown (shHMGCS1) on A549 cell migration and invasion (*n* = 4). **(F, G)** Subcutaneous injection of FOXA3-overexpressed LLC to C57 mice to induce lung cancer metastasis, HE staining **(F)**, and mRNA expression analysis **(G)** of lung tissues (*n* = 6). **(H, I)** The mice were subcutaneous injected with HMGCS1-knockdown LLC to induce lung cancer metastasis, HE staining **(H)**, and mRNA expression analysis **(I)** of lung tissues (*n* = 6). Scale bar, 50 μm **(C**, **D**, **G**, **H)**. Data were presented as mean ± SEM. *, *P* < 0.05; **, *P* < 0.01. HE, hematoxylin and eosin staining. The data underlying this figure can be found in the Supporting information file S1 Data.
(TIF)

S6 Fig Related to Fig 6. **(A–D)** The mRNA (*n* = 3) **(A)** and protein levels **(B)** of metastatic gene program in control (NC) or FOXA3-overexpressed A549 cells with or without HMGCS1 knockdown (shHMGCS1), representative images (left) and quantification (right) of transwell assay examining the effect of FOXA3 with or without HMGCS1 knockdown (shHMGCS1) on A549 cell migration and invasion (*n* = 4) **(C)**, and Cholesterol and 7-DHC levels of FOXA3-overexpressed A549 cells with or without HMGCS1 knockdown (shHMGCS1) (*n* = 3) **(D)**. **(E)** Immunoblot analysis of EGFR and Erk activation in A549 cells treated with cholesterol with or without MβCD. Scale bar, 50 μm **(C)**. Data were presented as mean ± SEM. *, *P* < 0.05; **, *P* < 0.01. Con, Control; Chol, Cholesterol. The data underlying this figure can be found in the Supporting information file S1 Data.
(TIF)

S7 Fig Related to Fig 7. **(A–C)** The cell viability curve of A549 **(A**, **B)** and H441 **(C)** treated with different concentrations of magnolol. **(D**, **E)** qPCR analysis of dose-dependent effect of magnolol on mRNA level of Foxa1, Foxa2, Foxa3 of A549 **(D)** and H441 **(E)** cells (*n* = 4). **(F**, **G)** qPCR analysis of Foxa3, Hmgcs1, and EMT-related genes of FOXA3-knockdown A549 **(F)** and H441 **(G)** cells treated with magnolol (*n* = 4). **(H)** Cell viability of foxa3^hi^ PDO treated with 10 μm magnolol, 10 μm fluvastatin, or 10 μm cisplatin by ATP assay (*n* = 6). **(I–O)** qPCR analysis of Ki67, Foxa3, Hmgcs1, and EMT-related genes in foxa3^hi^ PDO treated with 10 μm magnolol, 10 μm fluvastatin, or 10 μm cisplatin (*n* = 6). Data were presented as mean ± SEM. *, *P* < 0.05; **, *P* < 0.01. The data underlying this figure can be found in the Supporting information file S1 Data.
(TIF)

S8 Fig Related to Fig 7. Magnolol inhibits lung cancer progression in vivo. Mice were intravenously injected with A549-luc cells. Two weeks later, the mice were randomly assigned to the Con (Solvent control, *n* = 6) and Mag (15 mg/kg magnolol every other day, intraperitoneal injection, *n* = 6) groups for an additional 6 weeks of treatment. Subsequently, the mice underwent analysis through *IVIS* imaging **(A)**, qRT-PCR analysis of hGapdh/mGapdh in circulating tumor cells **(B)**, HE staining **(C)**, and qRT-PCR analysis of Foxa3, hmgcs1, and EMT-related genes **(D)**. Scale bar, 50 μm **(C)**. Data were presented as mean ± SEM. *, *P* < 0.05; **, *P* < 0.01. Con, DMSO; Mag, 10 μm magnolol; IVIS, In Vivo Imaging System; HE, hematoxylin and eosin staining; CTC, circulating tumor cells. The data underlying this figure can be found in the Supporting information file S1 Data.
(TIF)

S1 Table Top 30 enrichment genes of FOXA3 ChIP-seq dataset regarding binding gene name, location, and length. (DOCX)

S2 Table Top 20 compounds with inhibition rate of Foxa3 transcriptional activity. (DOCX)

S3 Table Primers used for plasmid constructions and real-time PCR. (DOCX)

S4 Table Primary antibodies. (DOCX)

S1 Data Individual numerical values corresponding to data presented in figures. (XLSX)

S1 Raw Images Raw images of data presented in Figs 2, 3, 4, 6, 7, and S1, S3, S4, S6. (PDF)

## Abbreviations

ChIP: chromatin immunoprecipitation
CPM: counts per million
CTC: circulation tumor cell
FPKM: fragments per kilobase of transcript per million fragments mapped
FOXA3: forkhead box A3
GO: Gene Ontology
HCC: hepatocellular carcinoma
IVIS: In Vivo Imaging Systems
KO: knockout
LDL: low-density lipoprotein
LDLR: low-density lipoprotein receptor
LPDS: lipoprotein-deficient serum
NSCLC: non-small-cell lung cancer
PBS: phosphate-buffered saline
PDO: patient-derived organoid
qRT-PCR: quantitative reverse transcription PCR
RLU: relative luciferase
SRA: Sequencing Read Archive
TC: total cholesterol
TCGA: The Cancer Genome Atlas
WT: wild-type
