# Supplementary figures and images for: FOXA3 regulates cholesterol metabolism to compensate for low uptake during the progression of lung adenocarcinoma
Source: PLoS Biol. 2024 May 28;22(5):e3002621. doi: 10.1371/journal.pbio.3002621 (PMC11161053; doi:10.1371/journal.pbio.3002621)

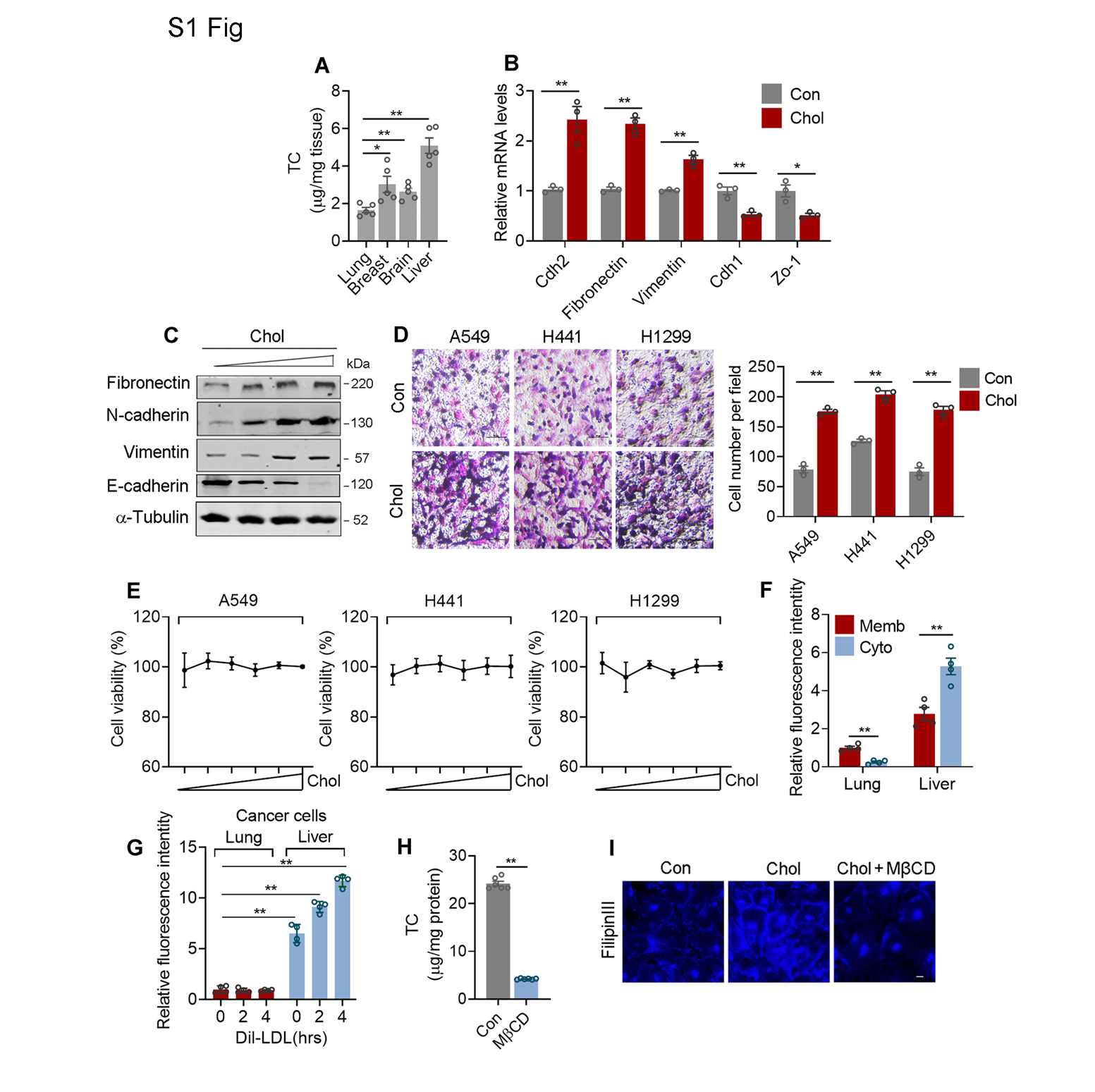

Supplement: S1 Fig — (A) The cholesterol levels in lung, breast, brain, and liver tissues of normal chow diet-fed mice (n = 5). (B, C) The mRNA (n = 3) (B) and protein (C) levels of metastatic genes in A549 cells cultured in LPDS medium and treated with 5 μg/ml cholesterol for 24 h. (D) Representative images (left) and quantification (right) of transwell assay examining the effect of exogenous cholesterol on cell migration of A549 cells (n = 3). (E, F) The effects of cholesterol on lung cancer cells viability. (F, G) Quantitative analysis of Fig 1E–1G were conducted using Image J (n = 4). (H) The cholesterol levels of A549 cells treated with 5 μg/ml cholesterol and/or MβCD for 24 h (n = 6). (I) The intracellular cholesterol levels detected by Filipin III in A549 cells treated with 5 μg/ml cholesterol for 24 h with or without MβCD pretreatment. Scale bar, 50 μm (D, I). Data were presented as mean ± SEM. *, P < 0.05; **, P < 0.01. Con, control; Chol, Cholesterol; Memb, Membrane; Cyto, Cytosol. The data underlying this figure can be found in the Supporting information file S1 Data. (TIF) [file pbio.3002621.s001.tif]

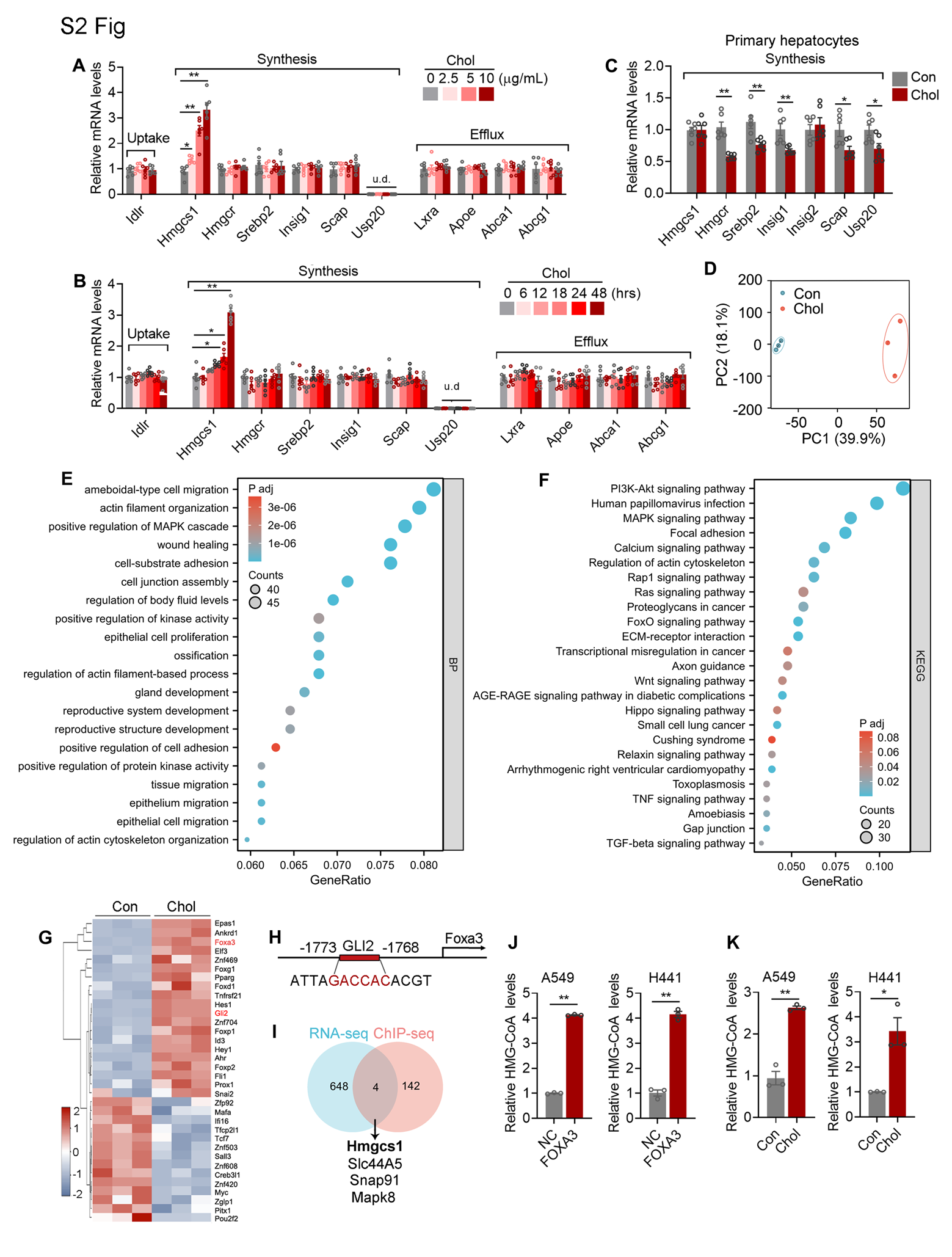

Supplement: S2 Fig — (A, B) Dose and time-dependent qPCR analysis of the mRNA levels of key cholesterol metabolism-related genes in cholesterol-treated A549 cells. (C) qPCR analysis of the mRNA levels of key cholesterol synthesis genes in primary hepatocytes (n = 6). (D–G) PCA of genes (D), GO-BP (E), and KEGG (F) and heat map of main transcriptional factors (G) of RNA-seq of A549 cells treated with or without cholesterol. (H) In silico analysis of putative GLI2 binding site on FOXA3 promoter. (I) Venn graph of the DEGs of RNA-seq and enriched genes of ChIP-seq. (J) The HMG-CoA levels in control (NC) or FOXA3 overexpressed A549 (left) or H441 (right) cells (n = 3). (K) The HMG-CoA levels in A549 (left) or H441 (right) cells treated with or without 5 μg/ml cholesterol in LPDS medium (n = 3). Data were presented as mean ± SEM. *, P < 0.05; **, P < 0.01. Con, control; Chol, Cholesterol; principal component analysis; DEG, differentially expressed genes. The data underlying this figure can be found in the Supporting information file S1 Data. (TIF) [file pbio.3002621.s002.tif]

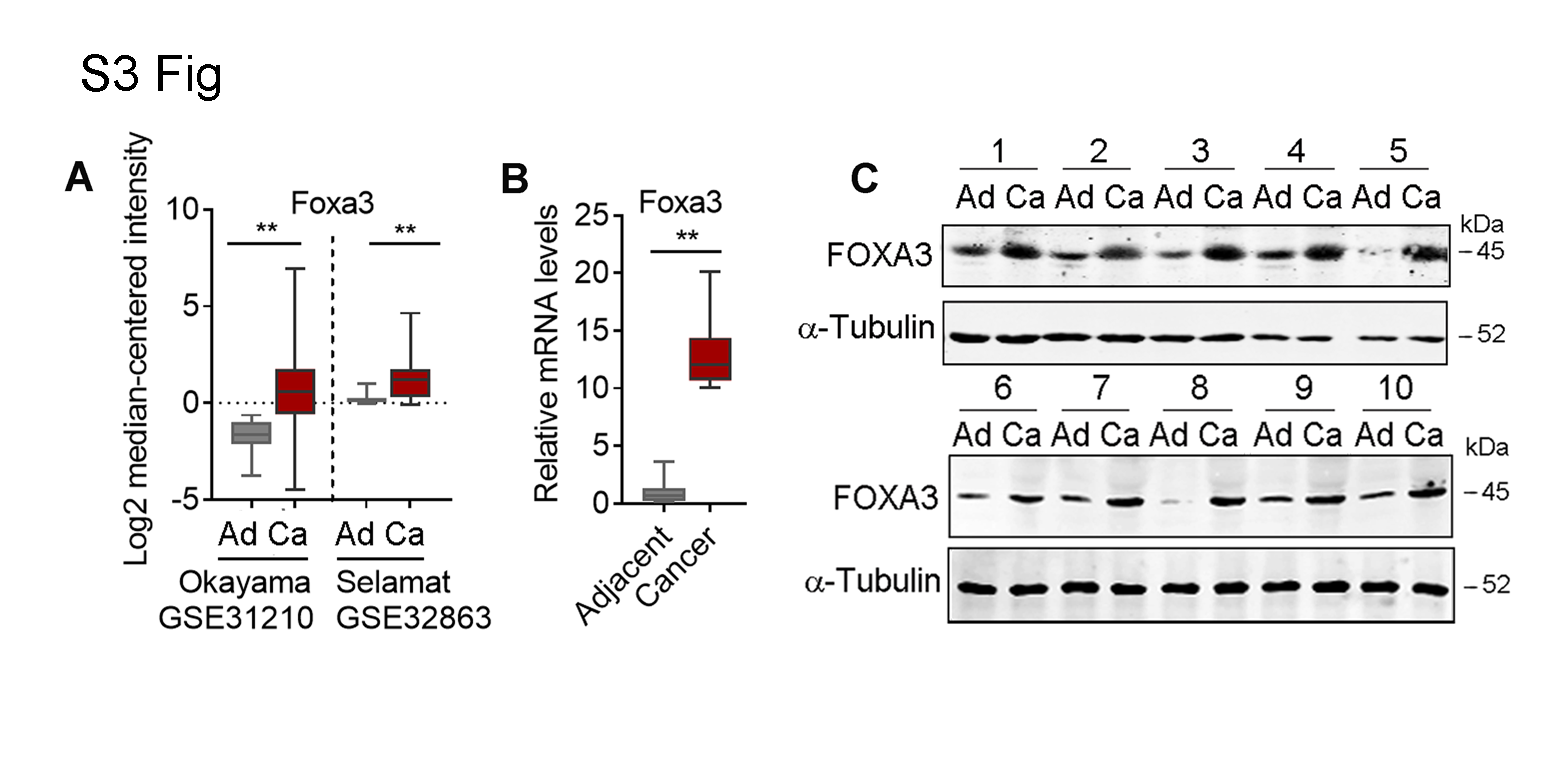

Supplement: S3 Fig — (A) Scatter diagram of Foxa3 levels in lung adenocarcinoma and adjacent tissues from oncomine database. (B, C) Foxa3 mRNA (B) and protein (C) levels in lung adenocarcinoma and adjacent tissues (n = 10 per group). Data were presented as mean ± SEM. *, P < 0.05; **, P < 0.01. Ad, Adjacent tissues; Ca, lung cancer tissues. The data underlying this figure can be found in the Supporting information file S1 Data. (TIF) [file pbio.3002621.s003.tif]

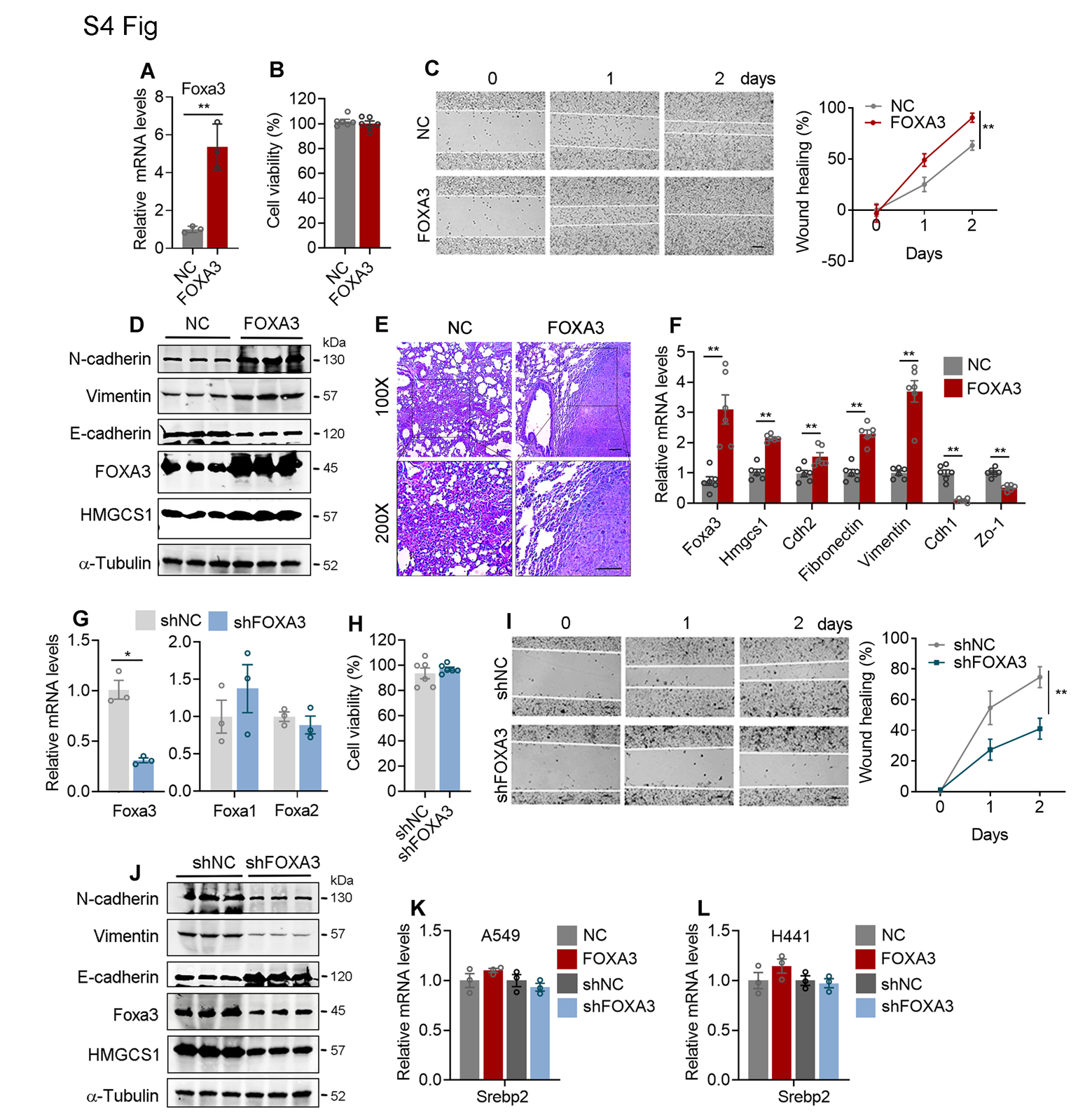

Supplement: S4 Fig — (A, B) Foxa3 mRNA levels (n = 3) (A) and cell viability (n = 6) (B) in control (NC) or Foxa3 overexpressed A549 cells. (C) Representative images (left) and quantification (right) of wound healing assay examining the effect of FOXA3 overexpression on A549 cell migration. (D) Immunoblotting analysis of HMGCS1 and EMT-related proteins in lung tissues of nude mice injected with FOXA3-overexpressed A549 cells. (E, F) Subcutaneous injection of FOXA3-overexpressed LLC in a lung cancer metastasis model, HE staining (E), mRNA expression analysis (F) of lung tissues (n = 6). (G, H) FOXA family members mRNA levels (n = 3) (G) and cell viability (n = 6) (H) in control (shNC) or Foxa3 knockdown (shFOXA3) A549 cells. (I) Representative images (left) and quantification (right) of wound healing assay examining the effect of FOXA3 knockdown on A549 cell migration. (J) Immunoblotting analysis of HMGCS1 and EMT-related proteins in lung tissues of nude mice injected with FOXA3-knockdowned A549 cells. (K, L) Srebp2 mRNA levels of FOXA3-overexpressed and FOXA3-knockdowned A549 (K) and H441 (L) cells (n = 3). Scale bar, 200 μm (C, I), scale bar, 50 μm (E). Data were presented as mean ± SEM. *, P < 0.05; **, P < 0.01. The data underlying this figure can be found in the Supporting information file S1 Data. (TIF) [file pbio.3002621.s004.tif]

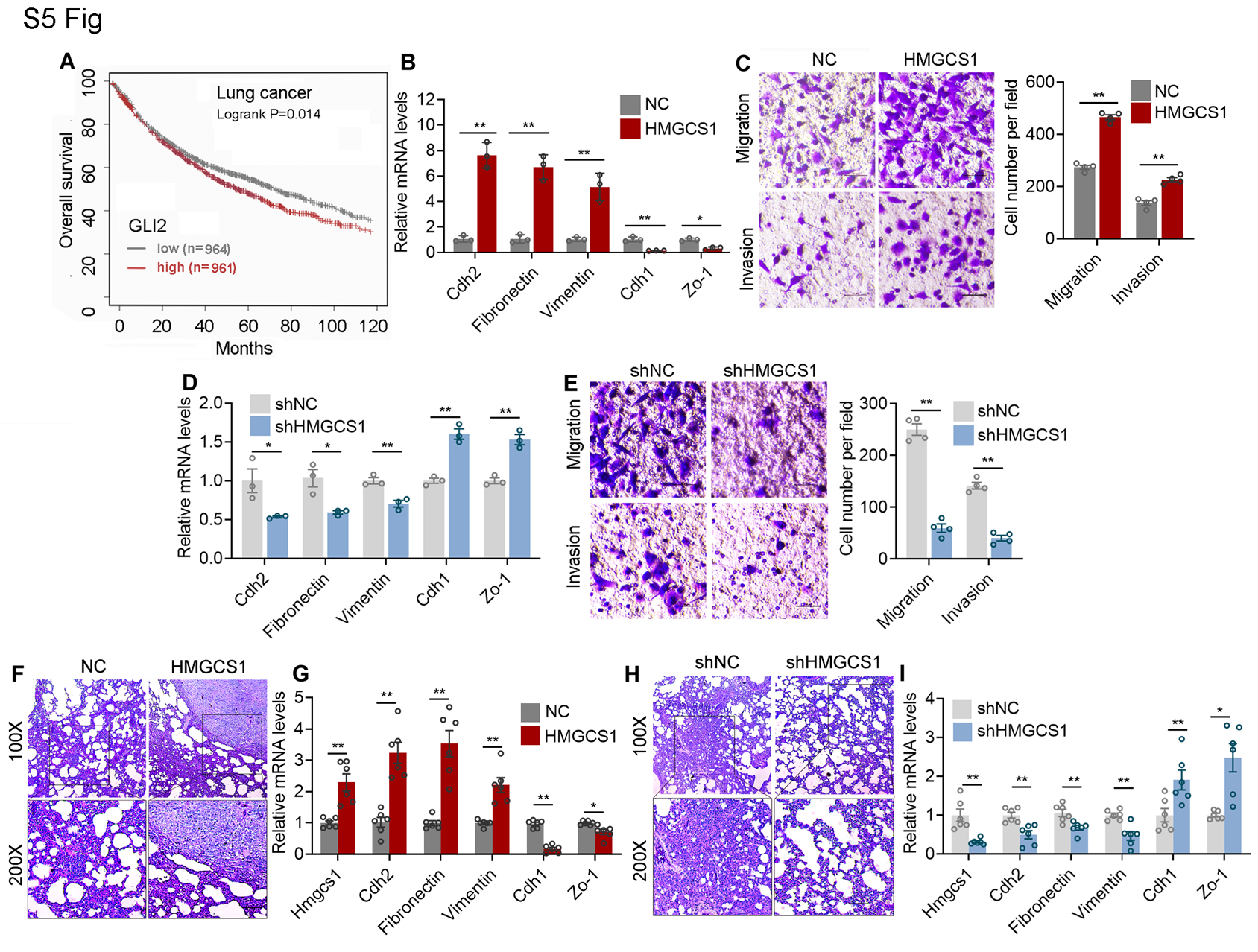

Supplement: S5 Fig — (A) Kaplan–Meier plotter showing the correlation between GLI2 levels and the overall survival of lung adenocarcinoma cancer patients from TCGA database (GLI2 low, n = 964; GLI2 high, n = 961). (B) qRT-PCR analysis of metastatic gene program in control (NC) or HMGCS1 overexpressed (HMGCS1) A549 cells (n = 3). (C) Representative images (left) and quantification (right) of transwell assay examining the effect of HMGCS1 overexpression on A549 cell migration and invasion (n = 4). (D) qRT-PCR analysis of metastatic gene program of control (shNC) and HMGCS1 knockdown (shHMGCS1) A549 cells (n = 3). (E) Representative images (left) and quantification (right) of transwell assay examining the effect of HMGCS1 knockdown (shHMGCS1) on A549 cell migration and invasion (n = 4). (F, G) Subcutaneous injection of FOXA3-overexpressed LLC to C57 mice to induce lung cancer metastasis, HE staining (F), and mRNA expression analysis (G) of lung tissues (n = 6). (H, I) The mice were subcutaneous injected with HMGCS1-knockdown LLC to induce lung cancer metastasis, HE staining (H), and mRNA expression analysis (I) of lung tissues (n = 6). Scale bar, 50 μm (C, D, G, H). Data were presented as mean ± SEM. *, P < 0.05; **, P < 0.01. HE, hematoxylin and eosin staining. The data underlying this figure can be found in the Supporting information file S1 Data. (TIF) [file pbio.3002621.s005.tif]

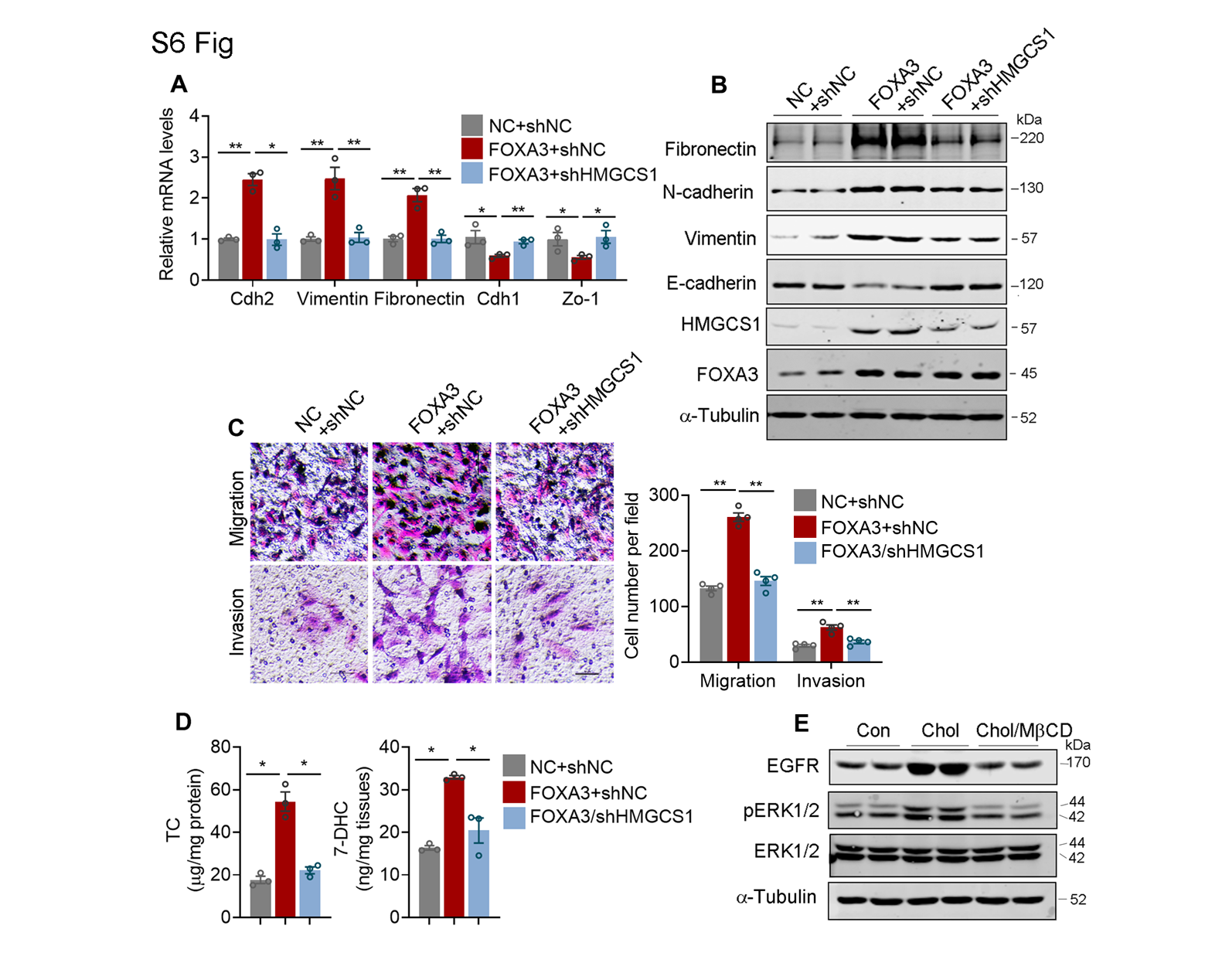

Supplement: S6 Fig — (A–D) The mRNA (n = 3) (A) and protein levels (B) of metastatic gene program in control (NC) or FOXA3-overexpressed A549 cells with or without HMGCS1 knockdown (shHMGCS1), representative images (left) and quantification (right) of transwell assay examining the effect of FOXA3 with or without HMGCS1 knockdown (shHMGCS1) on A549 cell migration and invasion (n = 4) (C), and Cholesterol and 7-DHC levels of FOXA3-overexpressed A549 cells with or without HMGCS1 knockdown (shHMGCS1) (n = 3) (D). (E) Immunoblot analysis of EGFR and Erk activation in A549 cells treated with cholesterol with or without MβCD. Scale bar, 50 μm (C). Data were presented as mean ± SEM. *, P < 0.05; **, P < 0.01. Con, Control; Chol, Cholesterol. The data underlying this figure can be found in the Supporting information file S1 Data. (TIF) [file pbio.3002621.s006.tif]

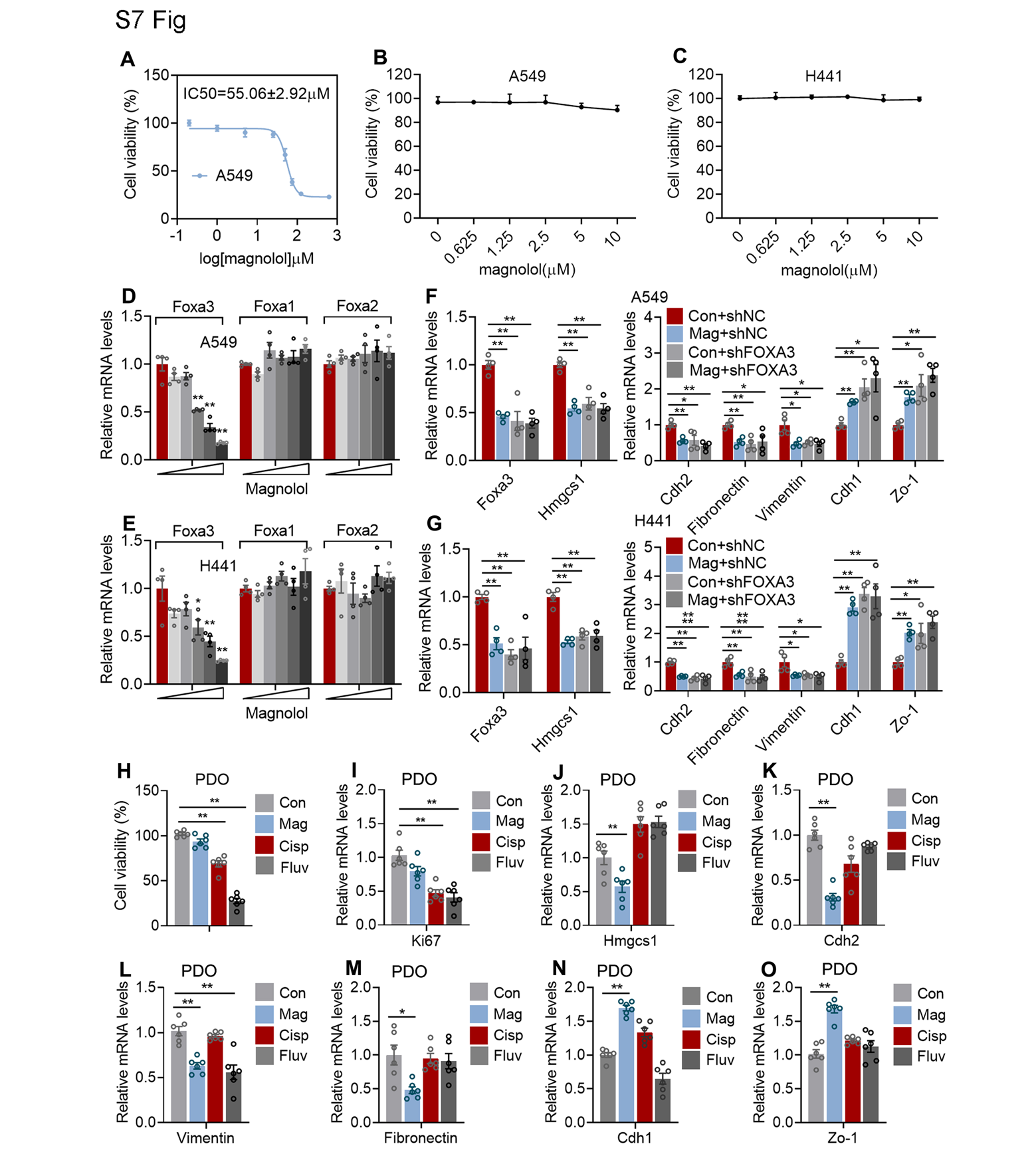

Supplement: S7 Fig — (A–C) The cell viability curve of A549 (A, B) and H441 (C) treated with different concentrations of magnolol. (D, E) qPCR analysis of dose-dependent effect of magnolol on mRNA level of Foxa1, Foxa2, Foxa3 of A549 (D) and H441 (E) cells (n = 4). (F, G) qPCR analysis of Foxa3, Hmgcs1, and EMT-related genes of FOXA3-knockdown A549 (F) and H441 (G) cells treated with magnolol (n = 4). (H) Cell viability of foxa3hi PDO treated with 10 μm magnolol, 10 μm fluvastatin, or 10 μm cisplatin by ATP assay (n = 6). (I–O) qPCR analysis of Ki67, Foxa3, Hmgcs1, and EMT-related genes in foxa3hi PDO treated with 10 μm magnolol, 10 μm fluvastatin, or 10 μm cisplatin (n = 6). Data were presented as mean ± SEM. *, P < 0.05; **, P < 0.01. The data underlying this figure can be found in the Supporting information file S1 Data. (TIF) [file pbio.3002621.s007.tif]

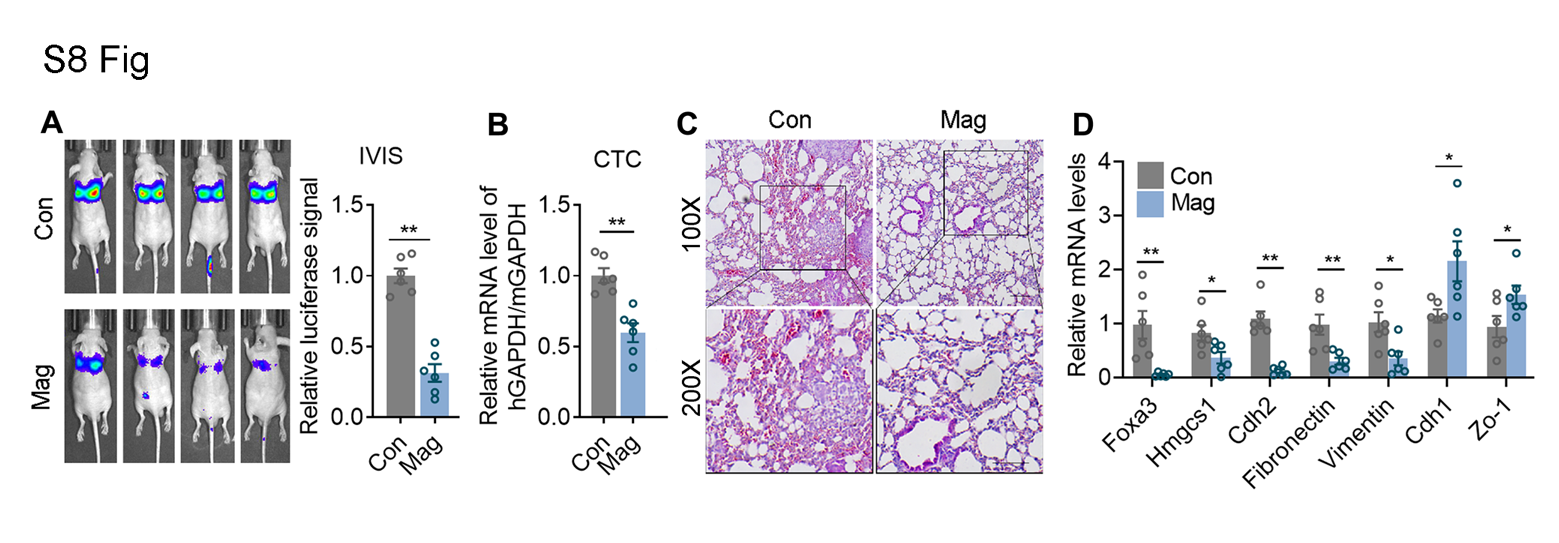

Supplement: S8 Fig — Magnolol inhibits lung cancer progression in vivo. Mice were intravenously injected with A549-luc cells. Two weeks later, the mice were randomly assigned to the Con (Solvent control, n = 6) and Mag (15 mg/kg magnolol every other day, intraperitoneal injection, n = 6) groups for an additional 6 weeks of treatment. Subsequently, the mice underwent analysis through IVIS imaging (A), qRT-PCR analysis of hGapdh/mGapdh in circulating tumor cells (B), HE staining (C), and qRT-PCR analysis of Foxa3, hmgcs1, and EMT-related genes (D). Scale bar, 50 μm (C). Data were presented as mean ± SEM. *, P < 0.05; **, P < 0.01. Con, DMSO; Mag, 10 μm magnolol; IVIS, In Vivo Imaging System; HE, hematoxylin and eosin staining; CTC, circulating tumor cells. The data underlying this figure can be found in the Supporting information file S1 Data. (TIF) [file pbio.3002621.s008.tif]
