# Supplementary material for: FOXA3 regulates cholesterol metabolism to compensate for low uptake during the progression of lung adenocarcinoma
Source: PLoS Biol. 2024 May 28;22(5):e3002621. doi: 10.1371/journal.pbio.3002621 (PMC11161053; doi:10.1371/journal.pbio.3002621)
Supplement: S1 Table — (DOCX) [file pbio.3002621.s009.docx]

**S1 Table. Top 30 enrichment genes of FOXA3 ChIP-seq dataset regarding binding gene name, location and length**

| **No.** | **Gene** | **fold**  **enrichment** | **chr** | **start** | **end** | **length** |
| --- | --- | --- | --- | --- | --- | --- |
| 1 | HMGCS1 | 6.70633 | chr5 | 43313485 | 43314193 | 709 |
| 2 | LRRFIP1 | 5.62279 | chr2 | 238691358 | 238691499 | 142 |
| 3 | SNTG2 | 5.58861 | chr2 | 1216015 | 1216161 | 147 |
| 4 | DCHS2 | 5.58861 | chr4 | 155230375 | 155230529 | 155 |
| 5 | ENPP2 | 5.5161 | chr8 | 120587100 | 120587258 | 159 |
| 6 | NOS1 | 5.49797 | chr12 | 117702903 | 117703075 | 173 |
| 7 | TMEM132B | 5.47997 | chr12 | 125946501 | 125946652 | 152 |
| 8 | GRB10 | 5.33994 | chr7 | 50671800 | 50672053 | 254 |
| 9 | FLJ33360 | 5.25616 | chr5 | 6326097 | 6326242 | 146 |
| 10 | TMEM220-AS1 | 5.22334 | chr17 | 10690977 | 10691120 | 144 |
| 11 | ADAM6 | 5.21198 | chr14 | 106435952 | 106436152 | 201 |
| 12 | KCNIP4 | 5.19431 | chr4 | 21209985 | 21210120 | 136 |
| 13 | LINC01492 | 5.19093 | chr9 | 105912917 | 105913055 | 139 |
| 14 | SLCO5A1 | 5.17585 | chr8 | 70662710 | 70662931 | 222 |
| 15 | ZNF709 | 5.11163 | chr19 | 12575468 | 12575654 | 187 |
| 16 | TENM4 | 5.04991 | chr11 | 78413695 | 78413884 | 190 |
| 17 | ZSWIM5 | 5.02975 | chr1 | 45628986 | 45629138 | 153 |
| 18 | NOS1 | 5.02975 | chr12 | 117667511 | 117667683 | 173 |
| 19 | NFATC2 | 5.02975 | chr20 | 50033284 | 50033487 | 204 |
| 20 | GABRA2 | 5.02975 | chr4 | 46252402 | 46252541 | 140 |
| 21 | FRMD3 | 5.02975 | chr9 | 85865884 | 85866131 | 248 |
| 22 | CCR6 | 5.00287 | chr6 | 167541464 | 167541649 | 186 |
| 23 | ZRANB3 | 4.99744 | chr2 | 136200461 | 136200621 | 161 |
| 24 | SLC35F1 | 4.99744 | chr6 | 118365186 | 118365334 | 149 |
| 25 | WDR70 | 4.94818 | chr5 | 37567858 | 37568003 | 146 |
| 26 | LRFN2 | 4.93197 | chr6 | 40395485 | 40395631 | 147 |
| 27 | LINC01258 | 4.90509 | chr4 | 38432904 | 38433058 | 155 |
| 28 | LINC01310 | 4.88398 | chr22 | 49267641 | 49267831 | 191 |
| 29 | LOC102546299 | 4.88398 | chr5 | 163963428 | 163963564 | 137 |
| 30 | CPED1 | 4.83691 | chr7 | 120905712 | 120905900 | 189 |
