# Supplementary material for: FOXA3 regulates cholesterol metabolism to compensate for low uptake during the progression of lung adenocarcinoma
Source: PLoS Biol. 2024 May 28;22(5):e3002621. doi: 10.1371/journal.pbio.3002621 (PMC11161053; doi:10.1371/journal.pbio.3002621)
Supplement: S2 Table — (DOCX) [file pbio.3002621.s010.docx]

**S2 Table. Top 20 compounds with inhibition rate of Foxa3 transcriptional activity**

| **No.** | **Compound(10μM)** | **Inhibition rate** | **p-value** |
| --- | --- | --- | --- |
| 1 | Magnolol (Mag) | 64.14±5.32% | 0.00343 |
| 2 | 6-Hydroxyflavone | 56.07±6.52% | 0.00669 |
| 3 | Sesamin | 52.44±7.06% | 0.00894 |
| 4 | Bufalin | 49.77±7.46% | 0.01104 |
| 5 | Icaritin | 48.20±7.69% | 0.01249 |
| 6 | Benzyl isothiocyanate | 47.46±7.8% | 0.01324 |
| 7 | Veratramine | 47.03±7.86% | 0.01369 |
| 8 | Trans-Anethole | 46.47±7.95% | 0.01431 |
| 9 | Digitoxin | 46.29±7.97% | 0.01452 |
| 10 | Dehydroandrographolide | 45.79±8.05% | 0.0151 |
| 11 | Osthole | 44.82±8.19% | 0.01629 |
| 12 | Genistein | 44.73±8.21% | 0.01641 |
| 13 | Pinostilbene | 44.45±8.25% | 0.01678 |
| 14 | Cinchonidine | 44.05±8.31% | 0.01732 |
| 15 | Catharanthine tartrate | 42.65±8.51% | 0.01935 |
| 16 | Phytic acid | 41.47±8.69% | 0.02124 |
| 17 | Schisandrin | 41.36±8.71% | 0.02145 |
| 18 | Bornyl acetate | 41.21±8.73% | 0.02169 |
| 19 | Stigmasterol | 40.27±8.87% | 0.02339 |
| 20 | Calcium folinate | 40.02±8.9% | 0.02388 |
