# Supplementary material for: FOXA3 regulates cholesterol metabolism to compensate for low uptake during the progression of lung adenocarcinoma
Source: PLoS Biol. 2024 May 28;22(5):e3002621. doi: 10.1371/journal.pbio.3002621 (PMC11161053; doi:10.1371/journal.pbio.3002621)
Supplement: S3 Table — (DOCX) [file pbio.3002621.s011.docx]

**S3 Table. Primers used for plasmid constructions and real-time PCR.**

| **Gene names** | **Forward/Reverse** | **Sequences 5'-3'** |
| --- | --- | --- |
| hFoxa3  (NM_004497.3) | Forward | GGTCTATTACTTACTGTGATGACTGCTG |
|  | Reverse | CCAAAGAAGATGTCACTGAAATGCT |
| hHmgcs1  (NM_001098272.3) | Forward | CATTAGACCGCTGCTATTCTGTC |
|  | Reverse | TTCAGCAACATCCGAGCTAGA |
| hHmgcr  (NM_000859.3) | Forward | TGATTGACCTTTCCAGAGCAAG |
|  | Reverse | CTAAAATTGCCATTCCACGAGC |
| hSrebp2  (NM_004599.4) | Forward | CCTGGGAGACATCGACGAGAT |
|  | Reverse | TGAATGACCGTTGCACTGAAG |
| hInsig1  (NM_005542.6) | Forward | GCCTACTGTACCCCTGTATCG |
|  | Reverse | TGGTTAATGCCAACAAAAACTGC |
| hScap  (NM_012235.4) | Forward | TATCTCGGGCCTTCTACAACC |
|  | Reverse | GGGGCGAGTAATCCTTCACA |
| hGapdh (NM_001256799) | Forward | CCACTGCCAACGTGTCAGTGGT |
|  | Reverse | CCCAGCAGTGAGGGTCTCTCTCT |
| hCdh1 (NM_001317184) | Forward | GGCACAGATGGTGTGATTACAGTC |
|  | Reverse | AGTCTCTCTTCTGTCTTCTGAGGCCA |
| hCdh2 (NM_001308176) | Forward | ATGCTGACGATCCCAATGC |
|  | Reverse | GCCTTCCATGTCTGTAGCTTGA |
| hVimentin (NM_003380.5) | Forward | GACGCCATCAACACCGAGTT |
|  | Reverse | CTTTGTCGTTGGTTAGCTGGT |
| hFibronectin  (NM_212482.3) | Forward | CGGTGGCTGTCAGTCAAAG |
|  | Reverse | AAACCTCGGCTTCCTCCATAA |
| hZo-1 (NM_003257.4) | Forward | CAACATACAGTGACGCTTCACA |
|  | Reverse | CACTATTGACGTTTCCCCACTC |
| hGli1  (NM_005269.3) | Forward | AGCGTGAGCCTGAATCTGTG |
|  | Reverse | CAGCATGTACTGGGCTTTGAA |
| hGli2  (NM_001371271.1) | Forward | CATGGAGCACTACCTCCGTTC |
|  | Reverse | CGAGGGTCATCTGGTGGTAAT |
| hGli3  (NM_000168.6) | Forward | TGGTTACATGGAGCCCCACTA |
|  | Reverse | GAATCGGAGATGGATCGTAATGG |
| hLdlr  (NM_000527.5) | Forward | ACCAACGAATGCTTGGACAAC |
|  | Reverse | ACAGGCACTCGTAGCCGAT |
| hFoxa1  (NM_004496.5) | Forward | GCAATACTCGCCTTACGGCT |
|  | Reverse | TACACACCTTGGTAGTACGCC |
| hFoxa2  (NM_021784.5) | Forward | GGAGCAGCTACTATGCAGAGC |
|  | Reverse | CGTGTTCATGCCGTTCATCC |
| hLxra  (NM_001130101.3) | Forward | TGGACACCTACATGCGTCGCAA |
|  | Reverse | CAAGGATGTGGCATGAGCCTGT |
| hApoe  (NM_000041.4 ) | Forward | GGGTCGCTTTTGGGATTACCTG |
|  | Reverse | CAACTCCTTCATGGTCTCGTCC |
| mHmgcr  (NM_001360165.1) | Forward | CTTGTGGAATGCCTTGTGATTG |
|  | Reverse | AGCCGAAGCAGCACATGAT |
| mSrebp2  (NM_001411792.1) | Forward | AGAAAGAGCGGTGGAGTCCTTG |
|  | Reverse | GAACTGCTGGAGAATGGTGAGG |
| mInsig1  (NM_153526.5) | Forward | ATAGCCACCATCTTCTCCTCCG |
|  | Reverse | CCAACGAACACGGCAATACAGC |
| mInsig2  (NM_001271531.1) | Forward | GTCAGTAGGACTGTGGTGGACT |
|  | Reverse | GGCAACCAAGAACGGACATAGAG |
| mScap  (NM_001001144.3 ) | Forward | GGAAATTGTCCTTCCGCCACTG |
|  | Reverse | CCGTGGATTCAGGTGTAGTGTG |
| mUsp20  (NM_028846.5) | Forward | TGGACTGCATAGGGGAGGTG |
|  | Reverse | ACTGGCAGGTTCCCTTAGATT |
| mFoxa3  (NM_008260.2) | Forward | CTACATGACCTTGAACCCACTC |
|  | Reverse | GGGCTACATACCCGGAAGC |
| mHmgcs1  (NM_145942.5) | Forward | GCCGTGAACTGGGTCGAA |
|  | Reverse | GCATATATAGCAATGTCTCCTGCAA |
| mGapdh (NM_001289726) | Forward | AGGTCGGTGTGAACGGATTTG |
|  | Reverse | GGCCTCACCCCATTTGATGT |
| mCdh1  (NM_009864.3) | Forward | CAGGTCTCCTCATGGCTTTGC |
|  | Reverse | CTTCCGAAAAGAAGGCTGTCC |
| mCdh2  (NM_007664.5) | Forward | AGCGCAGTCTTACCGAAGG |
|  | Reverse | TCGCTGCTTTCATACTGAACTTT |
| mVimentin  (NM_011701.4) | Forward | CGTCCACACGCACCTACAG |
|  | Forward | GGGGGATGAGGAATAGAGGCT |
| mFibronectin  (NM_010233.2) | Forward | ATGTGGACCCCTCCTGATAGT |
|  | Reverse | GCCCAGTGATTTCAGCAAAGG |
| mZo-1  (NM_009386.2) | Forward | GCCGCTAAGAGCACAGCAA |
|  | Reverse | TCCCCACTCTGAAAATGAGGA |
| ChIP Foxa3-promoter | Forward | \| GAGAACACAGTGGCGACAGA \| \| --- \| |
|  | Reverse | CCAGAAATCCGCCTGTTAAA |
| ChIP Foxa3-adjacent | Forward | GAACATTAGCTGGGCGTCTC |
|  | Reverse | CCAGAAATCCGCCTGTTAAA |
| ChIP Hmgcs1-promoter | Forward | TGCCTAATCCTGAAACCATTTTG |
|  | Reverse | GCAACCAGAACTAGGCTTCA |
| ChIP Hmgcs1-adjacent | Forward | GCTCCAATTTGCAATCTACGC |
|  | Reverse | GATGGTCAAATGTGGGAGGC |
| ChIP  β-globin | Forward | TTTAGTGATGGCCTGGCTCA |
|  | Reverse | ACCAGCCACCACTTTCTGAT |
| shFOXA3-1#  (NM_004497.3) | Forward | AACTTCAACCACCCTTTCTCC |
|  | Reverse | GGAGAAAGGGTGGTTGAAGTT |
| shFOXA3-2#  (NM_004497.3) | Forward | AATCTACCAGTGGATCATGGA |
|  | Reverse | TCCATGATCCACTGGTAGATT |
| shHMGCS1-1#  (NM_001098272.3) | Forward | TGTTGCCCTTGAGATCTATTT |
|  | Reverse | AAATAGATCTCAAGGGCAACA |
| shHMGCS1-2#  (NM_001098272.3) | Forward | GATCTTTCACTCACCATATTG |
|  | Reverse | CAATATGGTGAGTGAAAGATC |
| Foxa3 genotyping | Forward | GGCAGTGCTTCCGGGTATGTA |
|  | Reverse | GGGAAG AGGTCCATGATCCAT |
|  | LacZR | CAA AGCGCCATTCGCCATTCA |
