# Supplementary material for: FOXA3 regulates cholesterol metabolism to compensate for low uptake during the progression of lung adenocarcinoma
Source: PLoS Biol. 2024 May 28;22(5):e3002621. doi: 10.1371/journal.pbio.3002621 (PMC11161053; doi:10.1371/journal.pbio.3002621)
Supplement: S4 Table — (DOCX) [file pbio.3002621.s012.docx]

**S4 Table. Primary antibodies.**

| **Name** | **SOURCE** | **Catalog Number** |
| --- | --- | --- |
| FOXA3 antibody | Santa Cruz | sc-74424 |
| HMGCS1 antibody | Cell signaling | 42201 |
| E-cadherin antibody | BD biosciences | 610182 |
| N-cadherin antibody | BD biosciences | 610921 |
| Vimentin antibody | Abclonal | A19607 |
| Fibronectin antibody | Santa Cruz | sc-8422 |
| ɑ-Tubulin antibody | Santa Cruz | [sc-8035](https://www.scbt.com/zh/p/alpha-tubulin-antibody-tu-02?requestFrom=search) |
| Caveolin-1 antibody | Santa Cruz | sc-53564 |
| Normal mouse IgG | Santa Cruz | sc-2025 |
| Biotin antibody | Santa Cruz | sc-53179 |
| pErk1/2 antibody | Cell signaling | 9101 |
| Erk1/2 antibody | Cell signaling | 4695 |
| EGFR antibody | Cell signaling | 4267 |
