# Supplementary material for: FOXA3 regulates cholesterol metabolism to compensate for low uptake during the progression of lung adenocarcinoma
Source: PLoS Biol. 2024 May 28;22(5):e3002621. doi: 10.1371/journal.pbio.3002621 (PMC11161053; doi:10.1371/journal.pbio.3002621)

Raw images for Figure 2M and 2Q

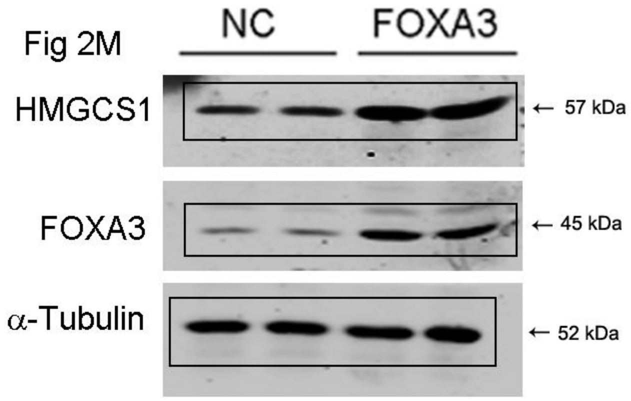

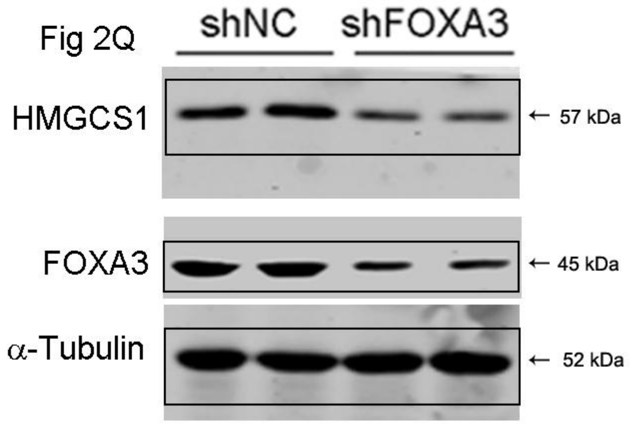

Raw images for Figure 3J

Fig 3J

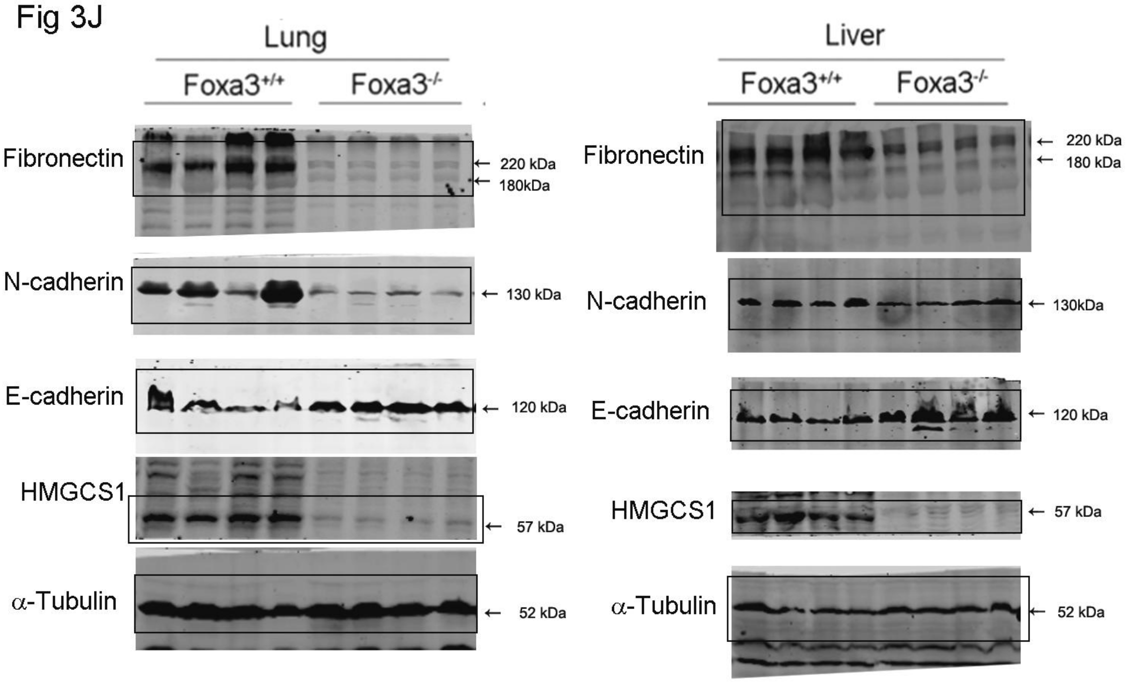

Raw images for Figure 4C and 4K

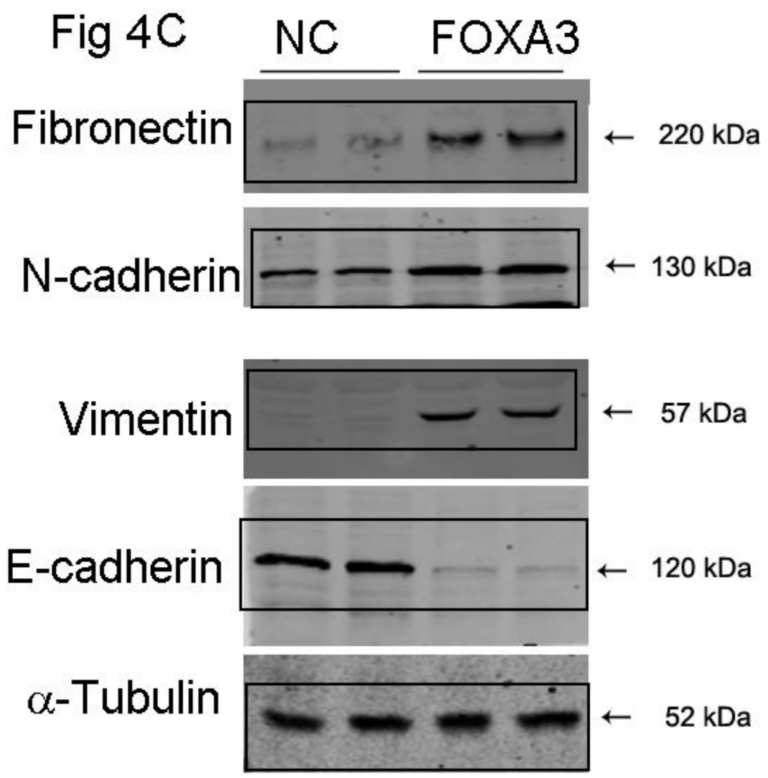

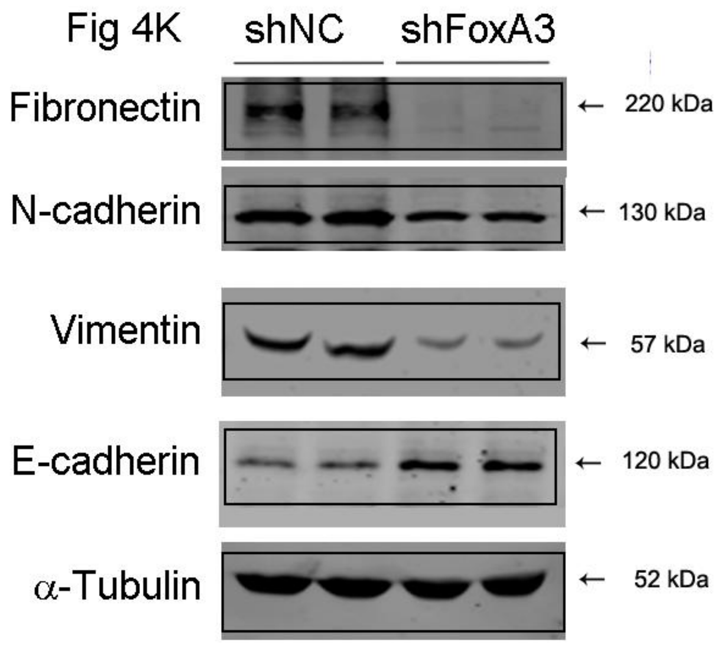

Raw images for Figure 6E, 6F, 6G, 6K

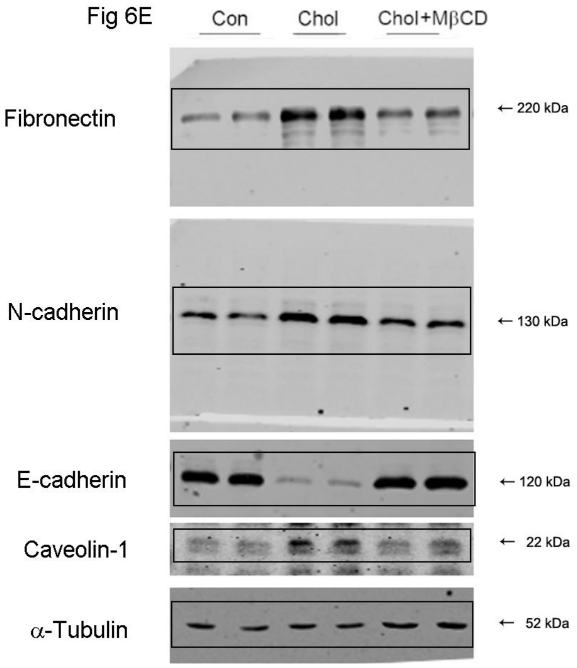

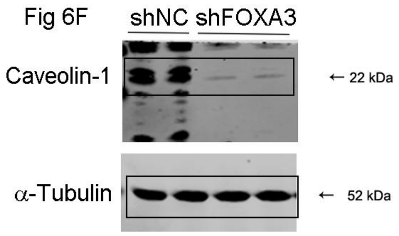

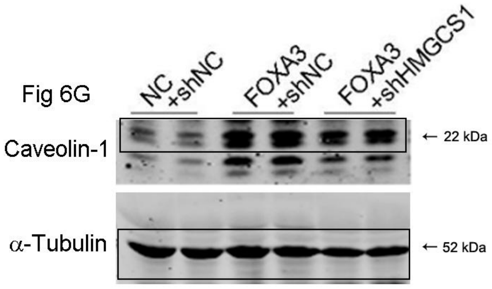

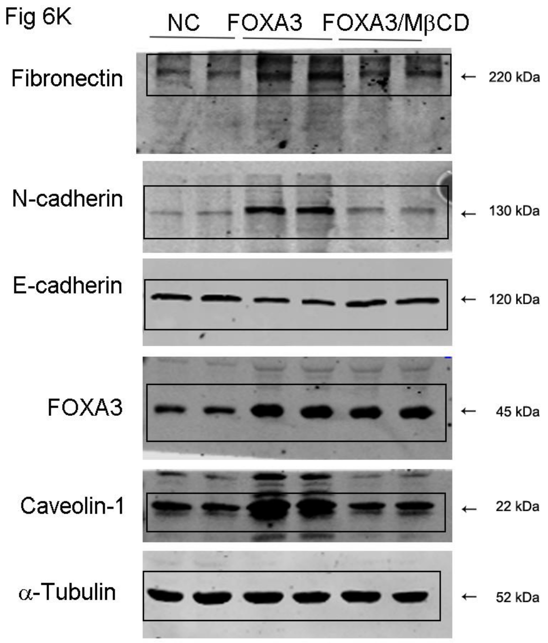

Raw images for Figure 7H

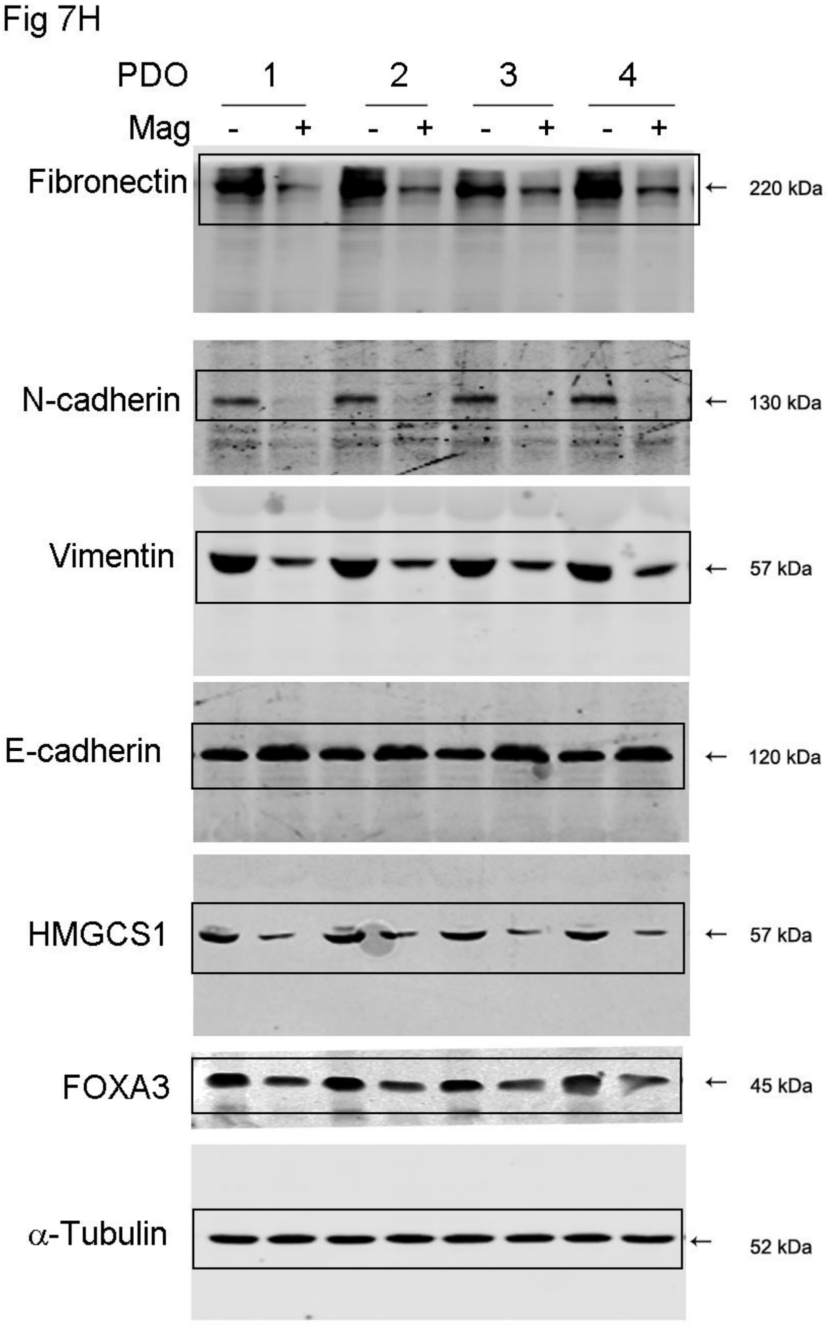

Raw images for Figure S1C

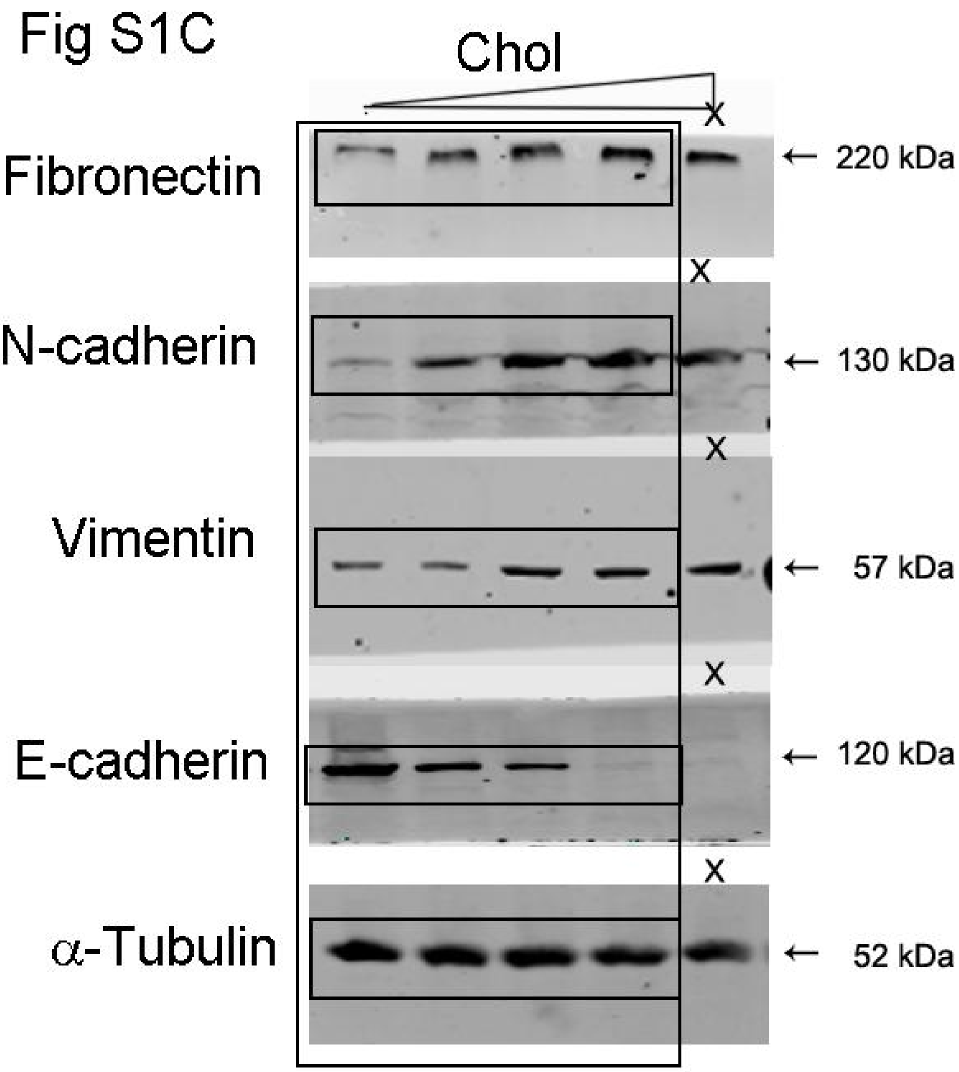

Raw images for Figure S3C

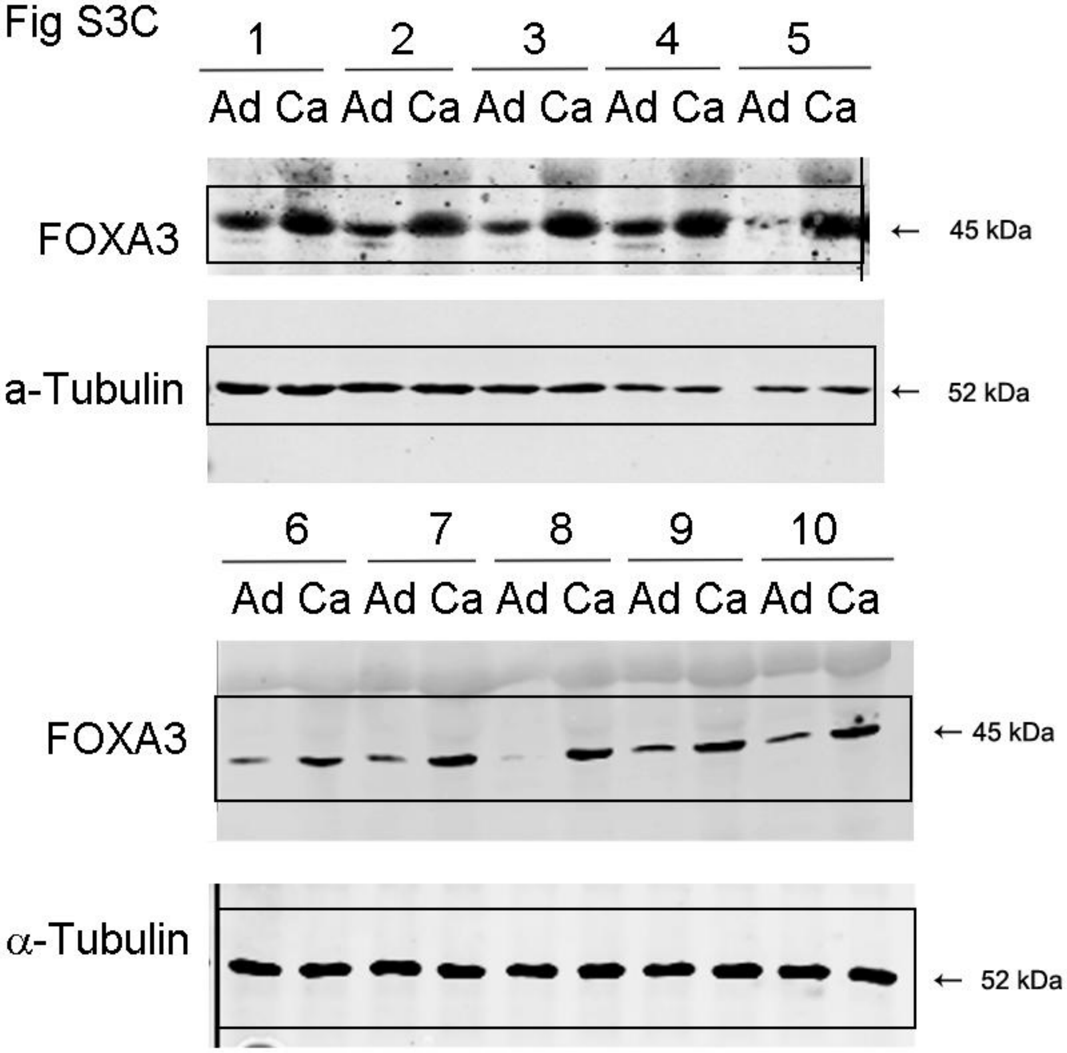

Raw images for Figure S4D and S4J

Fig S4D

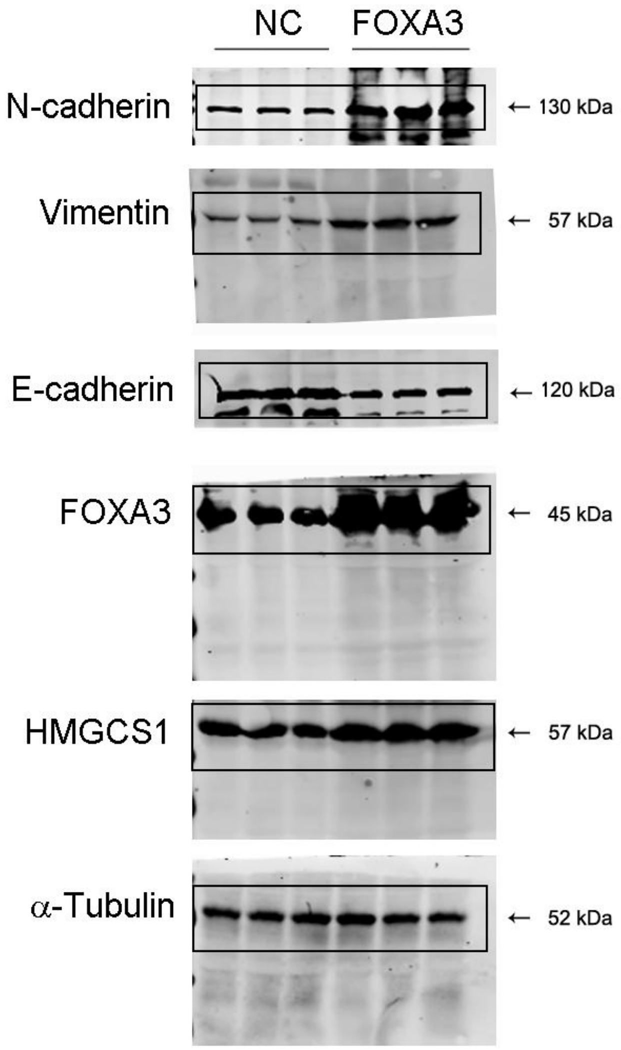

Fig S4J

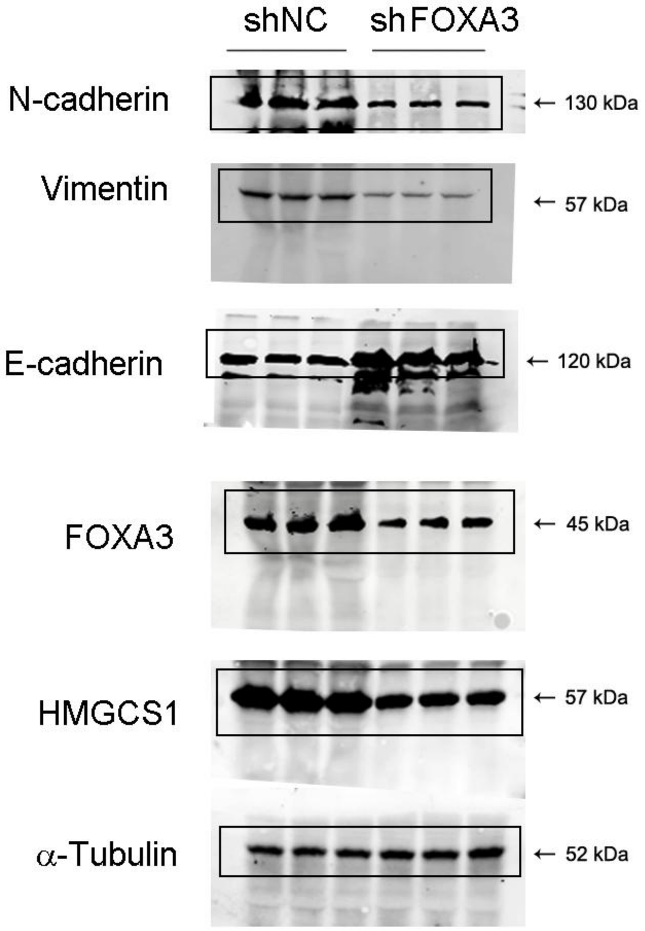

Raw images for Figure S6B and S6E

Fig S6B

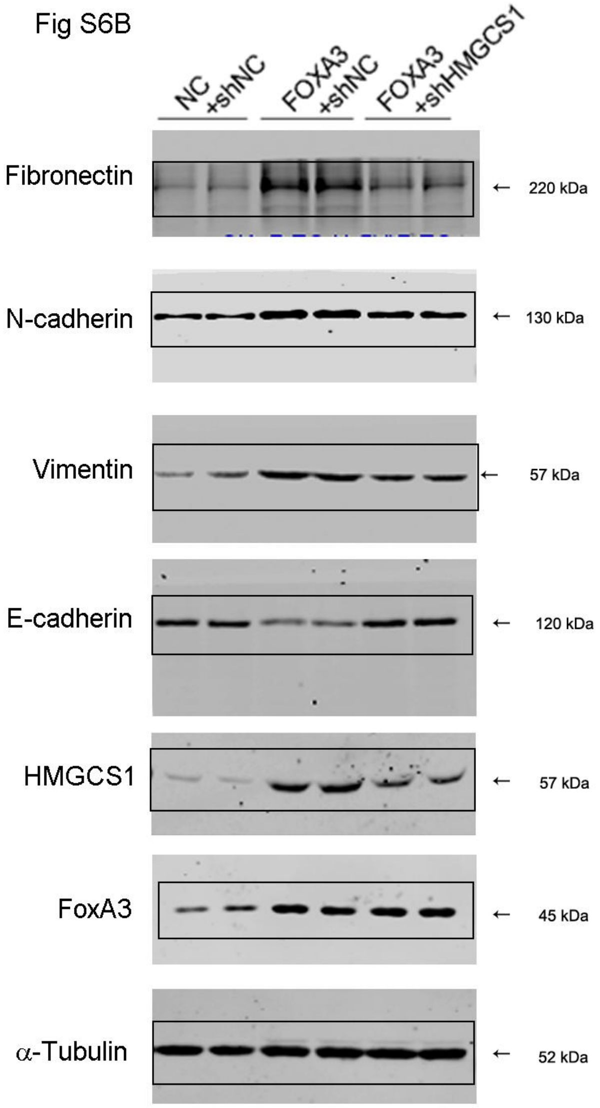

Fig S6E

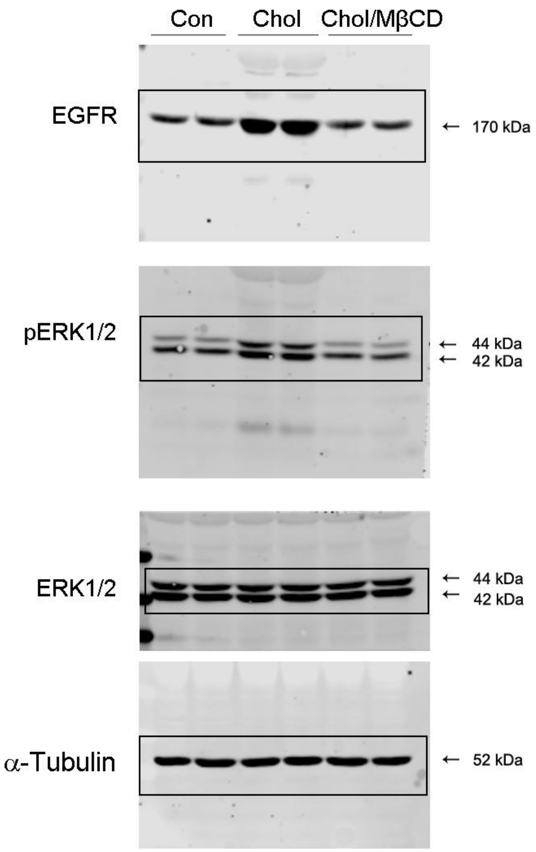

Supplement: S1 Raw Images — (PDF) [file pbio.3002621.s014.pdf]
